# Specifying the timescale of early life unpredictability helps explain the development of internalising and externalising behaviours

**DOI:** 10.1038/s41598-024-54093-x

**Published:** 2024-02-12

**Authors:** Bence Csaba Farkas, Axel Baptista, Mario Speranza, Valentin Wyart, Pierre Olivier Jacquet

**Affiliations:** 1 Institut du Psychotraumatisme de l’Enfant et de l’Adolescent, Conseil Départemental Yvelines et Hauts-de-Seine et Centre Hospitalier des Versailles, 78000 Versailles, France; 2 UVSQ, Inserm, Centre de Recherche en Epidémiologie et Santé des Populations, Université Paris-Saclay, 78000 Versailles, France; 3 LNC2, Département d’études Cognitives, École Normale Supérieure, INSERM, PSL Research University, 75005 Paris, France; 4 Centre Hospitalier de Versailles, Le Chesnay, France

**Keywords:** Psychology, Public health

## Abstract

Early life unpredictability is associated with both physical and mental health outcomes throughout the life course. Here, we classified adverse experiences based on the timescale on which they are likely to introduce variability in children’s environments: variations unfolding over short time scales (e.g., hours, days, weeks) and labelled *Stochasticity* vs variations unfolding over longer time scales (e.g., months, years) and labelled *Volatility* and explored how they contribute to the development of problem behaviours. Results indicate that externalising behaviours at age 9 and 15 and internalising behaviours at age 15 were better accounted for by models that separated Stochasticity and Volatility measured at ages 3 to 5. Both externalising and internalising behaviours were specifically associated with Volatility, with larger effects for externalising behaviours. These findings are interpreted in light of evolutionary-developmental models of psychopathology and reinforcement learning models of learning under uncertainty.

## Introduction

Early life adversity has lifelong consequences for both physical^1,2^ and mental health^3,4^. These effects are being increasingly understood from a dimensional perspective^5^. These conceptual models are based around the idea that, instead of assuming fully distinct or fully overlapping mechanisms for each adversity type (such as physical or sexual abuse, emotional neglect, poverty, etc.), the biological and psychological consequences of these experiences can be better accounted for by a set of core underlying dimensions. These dimensions cut through individual types of adversity experiences by focusing on the aspects of experience that are the most functionally relevant in calibrating development, and which can be found in many different individual adversity factors. Recent theoretical and empirical progress has integrated the Deprivation-Threat framework, rooted in experience-driven neuroplasticity^6,7^, and the Harshness-Unpredictability framework, rooted in behavioural ecology^8,9^, into a three-component model. This model is based on the assumption that Threat and Deprivation are best conceptualized as distinct sources of harshness, in the sense that both contribute to increasing disability and death in the population^10,11^. Three dimensions are identified: (i) Threat as a source of harshness capturing morbidity–mortality from harm imposed by other agents; (ii) Deprivation as a source of harshness capturing morbidity–mortality from insufficient environmental inputs; and (iii) Unpredictability as the spatiotemporal variations in both Threat and Deprivation. Although our understanding of the role played by these dimensions in the organization of human development has made important progress, we still know little about the impact of unpredictability. In this work, we aimed to explore how the timescale of unpredictability in early life environments channels psychological development through examining its unique or pervasive effect on internalizing (heightened anxiety, disturbed mood) and externalizing (conduct problems, heightened hyperactivity, heightened impulsivity) problem behaviours during childhood and adolescence.

Despite yielding consistent results, studies tended to operationalize unpredictability in very heterogeneous ways^12^. Diverse experiences, such as residential changes (e.g.^9^), subjective retrospective judgments of perceived unpredictability (e.g.^13^), inconsistent parenting (e.g.^14^), and family chaos (e.g.^15^) are all taken as indicators of the same general dimension. On the methodological side, the conceptual imprecision characterising the different methods used to measure early life unpredictability to date makes results difficult to compare across studies. On the theoretical side, there are multiple types of unpredictability with different statistical properties, each being possibly associated with a specific set of developmental responses. One feature of unpredictability that deserves a better specification is the period of time over which fluctuations actually impact predictability, i.e., its timescale^16,17^.

One reason why this question deserves attention is found in the conditions that favour the evolution of adaptive developmental plasticity. The developmental effects that are thought to reflect a kind of predictive adaptive response primarily emerge as the consequences of long rather than short timescale fluctuations of the environment^8–10,18^. This observation is supported by both modelling^18–20^ and empirical^17^ work, which collectively suggests that not all unpredictability signals should lead to adaptive phenotypic plasticity. Indeed, the rate of environmental change is a crucial determinant of the evolution of such predictive plasticity, and too high rates of fluctuations can prevent the possibility of adaptive phenotypic responses^21–23^. Children can therefore be expected to preferentially use early life experiences that fluctuate at a low to moderate rate as cues to predict the conditions under which they are likely to develop, and thus calibrate their phenotype accordingly.

The role of uncertainty timescales in generating adaptive responses has also been explored by the reinforcement learning literature^24,25^. Recent studies conducted in the laboratory on human learning have highlighted the crucial distinction between stochastic and volatile uncertainty^26,27^. *Stochasticity* refers to random fluctuations of environmental contingencies (e.g., reward outcomes) around a constant mean, whereas *volatility* is a genuine change in the mean itself. More concretely, day-to-day variability in the time it takes for you to arrive at work due to different traffic conditions would be an example of stochastic uncertainty. In contrast, a closing of the road you usually take due to road works, forcing you to take a different route would be volatile uncertainty, as in this case the mean travel time itself changes. Inferring this subtle distinction is quite crucial, as it determines the optimal response from the perspective of the learning agent. Stochasticity should not trigger significant learning as the best strategy is to average observed outcomes over a long time horizon and average out the ‘noise’. Volatility, on the other hand, should increase learning and adaptation, as the changed mean outcome must be inferred and adjusted to. In the reinforcement learning framework, this distinction corresponds to opposing optimal adjustments of learning rates. In one class of models, inspired by Kalman filters, learning rates are defined by the ratio of environmental stochasticity and volatility^26,27^. Humans have been shown to behave exactly this way in laboratory settings, i.e., downregulating learning rates when faced with stochasticity and upregulating learning rates when faced with volatility^28,29^. An intriguing question is whether the analogy can be made with developmental adaptations during childhood. To offer examples specific to childhood development, day-to-day variability in the time parents come back home would be an example of stochastic uncertainty, whereas an abrupt residential or school change would be volatile uncertainty, and one might expect adaptive developmental changes to occur more in response to the latter rather than the former uncertainty type.

Building on these theoretical frameworks and empirical observations, we hypothesize that there are at least two relatively independent timescales of variability in early life environments, each having distinct and coordinated effects on important developmental outcomes, i.e., the timing of reproductive effort and externalizing/internalizing behaviours. Here, we tentatively identify *stochasticity* with *short timescale*, day-to-day fluctuations that are relatively uninformative of environmental state changes, and *volatility* with *longer timescale* variability that signals meaningful changepoints.

To start investigating the descriptive utility of this distinction, we used data from the Fragile Families & Child Wellbeing Study, a longitudinal developmental cohort that follows children born to unmarried parents, in 20 large US cities^30^. We identify adverse experiences that introduce unpredictability on the day-to-day and week-to-week level (e.g., irregular bedtime) as short timescale (stochasticity), and experiences that introduce unpredictability on longer timescales (e.g., moving house) as long timescale (volatility). All major points of our methodology, including the creation of the environmental variables, model selection, data exclusion criteria, imputation methods and final sample size have all been pre-registered on the OSF platform (https://osf.io/3yxwt). We highlight any deviations from the pre-registration, and separate pre-registered and unregistered analyses.

We tested three main hypotheses: (i) Linear regression models separating the effects of stochastic and volatile unpredictability on internalising and externalising behaviours will provide a better fit, than models that contain an overall unpredictability term only. (ii) Stochasticity and volatility will show distinct patterns of associations with internalising and externalising behaviours. (iii) Stochasticity and volatility will interact in determining problem behaviours. We confirmed all 3 hypotheses and found that volatility seems to be uniquely related to the development of externalising behaviours. We followed up these initial results with an unregistered structural equation model, to see whether the effect of volatility is mediated by adolescent reproductive effort (here used as a proxy of individuals’ life history strategy) and found confirmatory evidence once again.

## Methods

### Sample

Data were drawn from the Fragile Families & Child Wellbeing Study (FFCWS). The FFCWS is a stratified, multistage sample of 4898 children born in 20 large U.S. cities between 1998 and 2000. FFCSW oversampled for nonmarital births and includes a large, diverse sample of children from lower income families and neighbourhoods, making it well suited for studying our research question. More details about the study and its methodology is available at the study website (https://fragilefamilies.princeton.edu/documentation), as well as in Reichman et al.^30^.

Data from all waves was accessed and used in the construction of some variables, however the main focus is on waves 3, 4, 5 and 6, covering focal child ages 3, 5, 9, and 15, respectively. All participants provided informed consent to participate, and all procedures were approved by the affiliated human subjects review board.

### Ethics declarations

All participating parents provided informed consent to participate, and all procedures were approved by the participating hospitals institutional review boards. All methods were performed in accordance with the relevant regulations and guidelines, and in accordance with the Declaration of Helsinki. For more information, see the study website (https://fragilefamilies.princeton.edu/documentation) as well as Reichman et al.^30^.

### Measures used in the pre-registered analyses

We were interested in capturing 4 dimensions of early life environments: Deprivation, Threat, Stochasticity and Volatility. Using linear regression models of various levels of complexity, we wished to investigate the associations between proxies of these 4 dimensions observed at ages 3 and 5, and our outcome variables, i.e., internalising and externalising behaviours measured at ages 9 and 15. To this end, we constructed aggregated scores, with the aim of capturing the mean frequency of experiences that may have cued individuals in their estimation of the environmental dimensions mentioned. All scores were sums of z-scored indicators. We now detail the creation of these scores, separately for our 4 dimensions. We based our construction of the Deprivation and Threat scores on the study of Miller et al.^31^. In the construction of the unpredictability scores, we aimed to sort indicators according to the conceptual criterion of whether they were likely to introduce unpredictability and uncertainty about expectations in the day-to-day and week-to-week timescale (Stochasticity), or on longer timescales (Volatility).

Our approach of constructing sum of z-scores composites was motivated by both theoretical reasoning and earlier empirical work. We considered that in the prevailing dimensional models of adversity we draw upon here, the dimensions are not defined by clustering of individual adversity factors, but by the nature of the experiences and their mechanistic effects on development. That is, adversity factors that comprise Deprivation or Threat or Unpredictability are not necessarily correlated with one another, instead they independently contribute to children’s expectations about their environment and influence their developmental trajectories. Our simple method of using sums of z-scored indicators has been used by previous work to model these types of emergent variables^8,32^. In such scores, more dispersed indicators (rarer adverse events) are effectively given a larger weight.

We also sought to confirm the distinct nature of these construct—and our composite scores aimed at approximating them—by a more data-driven approach. We reasoned that our composite scores obtained from the theoretical classification of adversity items into Deprivation or Threat on the one hand, and Volatility or Stochasticity on the other, should be more weakly correlated with each other than if they were constructed in a completely random and atheoretical manner. To verify it, we applied a permutation-based analysis in order to determine whether the correlation between the theory-driven Threat and Deprivation scores on the one hand, and the theory-driven Stochasticity and Volatility scores on the other hand, were indeed lower than the correlations observed between their ‘random-driven’ counterparts. As we report below in more details, results support the view of Threat and Deprivation as two relatively distinct and independent dimensions of adversity, and Stochasticity and Volatility as two relatively distinct and independent dimensions of unpredictability.

#### Deprivation

The Deprivation score was a sum of *Activities*, *Toys and books*, and *Parent–child interactions* indicators.

##### Activities

At ages 3 and 5, mothers were asked how many days per week they engage in a set of activities (e.g., sing nursery rhymes, play imaginary games) with the child (13 activities at age 3, 8 activities at age 5). For each activity, if an individual engaged in it more frequently than the sample mean, it was coded as a 0, otherwise as a 1. Then for each age, these scores were summed. These were z-scored to equalize scale and averaged across the two ages to create a single Activities indicator.

##### Toys and books

At ages 3 and 5, mothers were asked how many books and toys of various kinds does their child have (8 items in both ages). In each case, the response scale was between 1 and 4, indicating frequencies. Items were reverse coded such that greater scores would reflect greater deprivation and summed. These were z-scored to equalize scale and averaged across the two ages to create a single Toys and books indicator.

##### Parent–child interactions

At ages 3 and 5, during an in-home visit, observers indicated whether they observed a set of positive parent–child interactions during the visit (16 interactions at age 3, 17 interactions at age 5). For each item, responses were either 1 for present, or 0 for not present. When necessary, items were recoded such that greater scores reflected greater Deprivation and summed. These were z-scored to equalize scale and averaged across the two ages to create a single Parent–child interactions indicator.

#### Threat

The Threat score was a sum of *Psychological aggression*, *Physical aggression*, and *Community violence indicators*.

##### Psychological aggression

At ages 3 and 5, mothers completed the parent–child conflict tactics scales (CTSPC) measure^33^, which is aimed at measuring the presence and severity of parenting behaviours related to various subtypes of child maltreatment, corporal punishment, physical abuse, sexual abuse, psychological aggression, neglectful behaviour, and nonviolent discipline. To indicate psychological aggression, we used the psychological aggression subscale. Thus, we gave each response a value between 0 and 6, higher values indicating higher frequency, and summed each item. Scores for the two ages were then averaged to create a single psychological aggression indicator, and were then z-scored.

##### Physical aggression

The exact same procedure was used for the physical aggression indicator, except that we made use of the Physical assault subscale.

##### Community violence indicators

At ages 3 and 5, mothers completed a set of questions inquiring about how frequently they were the victim of or saw various types of violence in the past year (e.g., get hit by someone, attacked with a weapon). The same set of 17 items were asked at both ages. For each item, they could respond on a scale ranging from 0 to 4, higher values indicating greater frequency. Scores for the two ages were then summed and averaged to create a single Community violence indicator, which was then z-scored.

#### Stochasticity

The Stochasticity score was a sum of *Arrangements*, *Regular bedtime*, and *Bedtime routine* indicators.

##### Arrangements

At ages 3 and 5, mothers were asked whether their child is being cared for by someone other than parents, and if so, how many different arrangements are they using overall. As most mothers reported arrangements ranging between 0 and 3, a new variable was created based on these two items, which ranged from 0 (only parental care) to 3 (3 or more different arrangements). Scores for the two ages were then averaged to create a single Arrangements indicator, which was then z-scored.

##### Regular bedtime

At ages 3 and 5, mothers were asked whether their child has a regular bedtime, and if so, how many days was the child put to bed at that time in the past week. A new variable was created based on these two items, which ranged from 1 (no regular bedtime) to 6 (regular bedtime 5 days per week). Scores for the two ages were then averaged to create a single Regular bedtime indicator, which was then z-scored.

##### Bedtime routine

At ages 3 and 5, mothers were asked whether their child has a bedtime routine, and if so, in how many days was that routine followed in the past week. A new variable was created based on these two items, which ranged from 1 (no bedtime routine) to 6 (bedtime routine followed 5 days per week). Scores for the two ages were then averaged to create a single Bedtime routine indicator, which was then z-scored.

What ties these indicators together is that they are experiences that disrupt the predictability of the environment on the micro scale, from day-to-day, or week-to-week.

#### Volatility

The Volatility score was a sum of *Separations*, *Moves*, *Jobs* and *Maternal depression change* indicators.

##### Separations

At ages 3 and 5, mothers were asked how many times they have been separated from their child for a week or more. As most mothers reported separation ranging between 0 and 3, this variable was recoded such that scores ranged from 0 (no separations) to 3 (3 or more separations). Scores for the two ages were then averaged to create a single Separations indicator, which was then z-scored.

##### Moves

At ages 3 and 5, mothers were asked whether and how many times they have moved since last interview. As most mothers reported moving between 0 and 3 times, a new variable was created based on these two items, which ranged from 0 (no moves) to 3 (3 or more moves). Scores for the two ages were then averaged to create a single Moves indicator, which was then z-scored.

##### Jobs

At ages 3 and 5, mothers were asked whether and the total number of jobs they had since the focal child was born. As most mothers reported job numbers between 0 and 3, this item was recoded so that it ranged from 0 (no jobs) to 3 (3 or more jobs). Scores for the two ages were then averaged to create a single Jobs indicator, which was then z-scored.

##### Maternal depression change

Based on the CIDI-SF depression criteria scores described below, a new variable was created which tracked whether the current depression criteria of the mother has changed since last interview (either from meeting criteria to not meeting criteria, or from not meeting criteria to meeting criteria). Scores for the two ages were then averaged to create a single Maternal depression change indicator, which was 0 if criteria changed at either year compared to last interview, 0.5 if they only changed at one of the years, and 1 if they changed at both years. This indicator was also z-scored before entering into the aggregated score.

What ties these indicators together is that they are experiences that disrupt the predictability of the environment on the macro scale, from month-to-month, or year-to-year.

#### Overall unpredictability

An overall unpredictability score was also created by simply summing all indicators of both stochastic and volatile unpredictability.

#### Internalising behaviours

Internalising behaviours were assessed using the total sum scores from the available items of the Child Behavior Checklist (CBCL^34^). For age 5, 72 items; for age 9, 111 items; and for age 15, 34 items were administered, all completed by the primary caregiver. We used the commonly used total internalising subscale, which comprises all items from the anxious/depressed, somatic complaints and withdrawn/depressed scales. For ages 5 and 15, the somatic complaints subscale was not available.

#### Externalising behaviours

Externalising behaviours were similarly assessed using the total sum scores from the available items of the CBCL. We used the commonly used total externalising subscale, which comprises all items from the aggressive and rule-breaking scales.

#### Socio-economic status

SES was assessed by the poverty ratio at ages 9 and 15. The poverty ratio is the ratio of total household income, to the official poverty thresholds, designated by the U.S. Census Bureau.

### Additional measures used in the unregistered analysis

#### Reproductive effort

For the unregistered structural equation model, we aimed to create a latent variable capturing individuals’ reproductive effort. For this, we used 4 indicators: Number of sexual partners, Number of date partners, Age at 1st sexual intercourse, and Age at 1st date. All of these indicators were assessed at age 15, and they were all z scored before entering into the models.

##### Number of sexual partners

Total number of lifetime sexual partners, self-reported by youth*.*

##### Number of date partners

Total number of persons ever dated, self-reported by youth*.*

##### Age at 1st sexual intercourse

Age when youth first had consented sexual intercourse, self-reported by youth*.* This variable was recoded into 4 ordered categories: 1 = earlier than age 13, 2 = between age 13 and 14, 3 = after age 14, 4 = no sexual intercourse yet.

##### Age at 1st date

Age when youth first dated someone, self-reported by youth*.* This variable was recoded into 4 ordered categories: 1 = earlier than age 12, 2 = between age 12 and 13, 3 = after age 13, 4 = not dated anyone yet.

### Missing data

We only used data from respondents who completed all waves of the FFCWS, which meant 2784 subjects. This is the sample that the frequency of missing values for each variable, presented in Supplementary Table S1, was evaluated on. From this sample, we further excluded subjects who had missing values on any of the variables we did not wish to impute (see below). This left us with a final sample size of 1642 for the age 9 regression models, 2218 for the age 15 regression models and 1730 for the age 15 structural equation model.

We tried to assess whether data were missing completely at random (MCAR) using two approaches. First, we explored patterns of missingness by pairwise comparisons (Kruskal–Wallis tests for continuous and Chi squared tests for discrete data) of our explanatory variables as a function of missingness for each of our 4 outcome variables (Supplementary Tables S6–S9). Although uncorrected p values indicated some systematic patterns, with somewhat higher indices of adversity for individuals with missing data on our outcome variables, none of these comparisons survived correction for multiple comparisons, using the Benjamini–Hochberg procedure^35^. Second, we calculated Little’s MCAR test^36^, which also failed to reject the null hypothesis that the data are MCAR, χ^2^_(17,666)_ = 7107, p = 0.999.

Missing data were thus handled using multiple imputation by chained equations, as implemented in the *mice* package in R. We used the default imputation methods, such that numerical variables were imputed using predictive mean matching, and ordered variables were imputed using a proportional odds model. Using this approach, 20 imputed datasets were created. We didn’t impute sex, mother depression criteria, the reproductive effort indicators and CBCL scores. We carried out the imputation on the raw indicator scores, before averaging across years and z-scoring.

### Pre-registered data analytic plan and deviations

The creation of the composite scores, and the data analytic plan were pre-registered at OSF (https://osf.io/3yxwt). Our pre-registered analytic plan is centred on multiple linear regressions on multiply imputed data. However, we deviate from our pre-registered methods in several respects. Firstly, we decided to add a model that includes an overall unpredictability composite, as comparing our models separating stochastic and volatile unpredictability to this overall unpredictability model is of crucial theoretical importance. However, this implied that the models were non-nested, so they could not be compared with the D3 likelihood-ratio test. Therefore, contrary to the pre-registration, we only rely on the Akaike Information Criterion (AIC^37^) for model comparisons, although we also report the Bayesian Information Criterion (BIC^38^) to allow comparisons between different model selection criteria. Moreover, comments and reviews on earlier versions of this manuscript and further reflection led us to simplify the models, such that Deprivation and Threat do not have interactive terms with Stochasticity and Volatility, and maternal depression is not included either as a part of the stochasticity composite or as a control variable. To sum up, we thus converged on 4 linear regression models of increasing complexity: a *Baseline* model, an *Overall unpredictability* model, a *Stochasticity, volatility main effects* model, and a *Stochasticity, volatility interaction* model. Deprivation, Threat, Sex, Problem behaviours at the previous assessment age and SES at the current assessment age are included as control variables in all models.

#### Linear regression models

These analyses were carried out in R version 4.2.3^39^, with the *mice* package^40^.

The following 4 models were run for each of the 4 outcome variables *Internalising behaviours at age 9*, *Externalising behaviours at age 9*, *Internalising behaviours at age 15*, *Externalising behaviours at age 15*.

##### Model 1

A *Baseline* model with only control variables of Deprivation, Threat, SES, Sex, and Behaviours at previous assessment:

$${\text{Y }} = {\text{ b}}_{0} + {\text{ b}}_{{1}} SES + {\text{ b}}_{{2}} Sex + {\text{ b}}_{{3}} Previousbehaviours + {\text{ b}}_{{4}} Deprivation + {\text{ b}}_{{6}} Threat.$$

##### Model 2

An *Overall unpredictability* model, which additionally contained a main effect of an unpredictability composite that did not separate stochastic and volatile unpredictability:

$${\text{Y }} = {\text{ b}}_{0} + {\text{ b}}_{{1}} SES + {\text{ b}}_{{2}} Sex + {\text{ b}}_{{3}} Previousbehaviours + {\text{ b}}_{{4}} Deprivation + {\text{ b}}_{{5}} Threat + {\text{ b}}_{{6}} Unpredictability.$$

##### Model 3

A *Stochasticity, volatility main effects* model, which contained the main effects of Stochasticity and Volatility:

$${\text{Y }} = {\text{ b}}_{0} + {\text{ b}}_{{1}} SES + {\text{ b}}_{{2}} Sex + {\text{ b}}_{{3}} Previousbehaviours + {\text{ b}}_{{4}} Deprivation + {\text{ b}}_{{5}} Threat + {\text{ b}}_{{6}} Stochasticity + {\text{ b}}_{{7}} Volatility.$$

##### Model 4

A *Stochasticity, volatility interaction* model that further contained an interaction term between Stochasticity and Volatility:

$$= {\text{ b}}_{0} + {\text{ b}}_{{1}} SES + {\text{ b}}_{{2}} Sex + {\text{ b}}_{{3}} Previousbehaviours + {\text{ b}}_{{4}} Deprivation + {\text{ b}}_{{5}} Threat + {\text{ b}}_{{6}} Stochasticity + {\text{ b}}_{{7}} Volatility + {\text{ b}}_{{8}} Stochasticity \, \times \, Volatility.$$

These models test our 3 main hypotheses:

###### Hypothesis 1

Models with separate terms for Stochasticity and Volatility will provide better fits than models containing only Deprivation and Threat, and Overall unpredictability, i.e., the best fitting model for all outcomes will be either Model 3 or 4.

###### Hypothesis 2

Stochasticity and Volatility will have distinct and specific associations with internalising and externalising behaviours, although because of the limited extant literature on this issue, we were agnostic about what specific associations to expect.

###### Hypothesis 3

Stochasticity and Volatility will interact with each other, i.e., the best fitting model for all outcomes will be Model 4. With respect to the interaction between Stochasticity and Volatility, again, due to the limited extant literature, we were agnostic as to what pattern to expect.

Plots of model residuals against fitted values, and Q–Q plots of standardized residuals for each of the 4 main models are reported in Supplementary Fig. S3.

#### Leave-one-indicator-out procedure

We also tested the robustness of our model specification by a leave-one-indicator-out procedure. For each effect that involved Stochasticity or Volatility, we took the scores that are included in the best fitting model, and refit the model multiple times, each time leaving out one indicator in the creation of a score. This sensitivity analysis lets us see whether one indicator heavily biases our results.

### Unregistered analyses

#### Structural equation model

There is ample empirical evidence for the association between dimensions of early life adversity and human life histories, which define variations in the way organisms organize their life schedule in order to optimize the achievement of their biological goals given a limited stock of bioenergetic resources^8,41^. In environments conferring large or unpredictable risk of death and illness, organisms tend to favour reproductive efforts and short-term goals, to the detriment of somatic maintenance efforts and longer-term goals. Importantly, this accelerated life history strategy is not only mediated by physiological mechanisms. It is also dependent on psychological traits facilitating priority goals, such as lower mate selectivity, sexual promiscuity, intrasexual competition for mates and status, and lower parenting effort^42^. It turns out that such dangerous and uncertain environments can contribute, via extreme levels of both physiological and psychological components of fast life history strategies, to a host of physical and mental health problems^9,43,44^. These effects are strongest for externalising behaviours, characterized by risk-taking, impulsivity and substance use. Engaging in such risky behaviours, especially during adolescence, can draw the attention of peers and increase reputation and social standing, thus facilitating related biological goals^45,46^. Thus, life history strategies can be important mediators of the effects of early life adversity on psychopathology, and incorporating data about fundamental life history trade-offs, such as between current and future reproductive effort can enhance the predictive and explanatory power of existing models. One would expect that early life adversity will have the strongest impact on externalising psychopathology in people who develop an accelerated life history strategy the most.

To explore this idea, in an unregistered, follow-up analysis to our main results, we aimed to test whether the effects of early life adversity dimensions on adolescent problem behaviours are mediated by reproductive effort. The trade-off between current and future reproduction is an important component of human life histories^47^, and early life adversity has been linked to earlier reproduction and higher reproductive effort^32,48,49^.

To this end, we fit structural equation models (SEM) using the lavaan^50^ and semTools^51^ packages in R. These models are made up of a ‘measurement’ model that relates the observed ‘indicators’ to hypothesised, but unobservable ‘latents’ and a ‘structural’ model that relates the latent variables to each other by specifying paths between them.

The models were fitted using a weighed least squares estimator (WLSMV) because of its robustness to deviations from normality. Model fit was assessed by the Comparative Fit Index (CFI), Root Mean Square Error of Approximation (RMSEA), and Standardized Root Mean Square Residual (SRMR) statistics. The CFI, RMSEA, and SRMR statistics were corrected by a scaling factor, which compensates for the average kurtosis of the data^52^. A model’s fit is generally considered excellent when the RMSEA is below 0.05, the CFI is above 0.95 and the SRMR is below 0.08^53^.

The measurement model of the Reproductive effort latent variable consisted of the Number of sexual partners, Number of date partners, Age at 1st sexual intercourse, and Age at 1st date indicators. These indicators were adjusted for Deprivation, Threat, SES at age 15, and sex. The structural model consisted of direct effects on age 15 internalising and externalising behaviours, of all 4 early life adversity dimension composites, and indirect effects through Reproductive effort. The problem behaviours were adjusted for the same set of covariates as our main regression models, that is, Deprivation, Threat, SES at age 15, sex, and problem behaviours at age 9. The covariance between internalising and externalising behaviours was also estimated. Based on earlier results, we expected that higher Volatility will lead to accelerated life courses, indexed by higher and earlier reproductive effort, which in our models would correspond to both earlier ages at 1st sexual intercourse and dating, and higher total number of sexual and dating partners.

## Results

Basic demographic information (sex and ethnic group) for the sample is presented in Table 1. There was an almost even split between men and women, the largest ethnic group in the sample was Black, followed by Hispanic, White, and Multi-racial. Correlations between all variables are presented in Supplementary Table S10. Briefly, Deprivation, Threat, Stochasticity, and Volatility were all weakly-to-moderately correlated with each other, and weakly positively correlated with internalising and externalising behaviours. Internalising and externalising behaviours were moderately-to-strongly correlated with each other. The distributions of all variables can be found in Supplementary Fig. S1 (most were long tailed). Fraction of missing data for each variable is presented in Supplementary Table S1.

**Table 1:** Sample descriptive statistics.

Total N	2218
Child’s sex	
Male	1137 (51.3%)
Female	1081 (48.7%)
Child’s self-described race	
White	401 (18.1%)
Black	1036 (46.7%)
Hispanic	471 (21.2%)
Other	56 (2.5%)
Multi-racial	100 (4.5%)
NA	154 (6.9%)
Maternal education	
Less than high school	646 (29.1%)
High school or equivalent	714 (32.2%)
Some college	581 (26.2%)
College or graduate	276 (12.4%)
NA	1 (0.1%)
Paternal education	
Less than high school	626 (28.2%)
High school or equivalent	801 (36.1%)
Some college	480 (21.6%)
College or graduate	241 (10.9%)
NA	70 (3.2%)
Age 3 poverty ratio	
Mean	2.04
SD	2.78

### Relative independence of threat/deprivation and volatility/stochasticity constructs

It is notable that the correlation between stochasticity and volatility was the lowest (r = 0.086) among all early life adversity dimensions, suggesting that the two timescales of variability are relatively independent. If this is true, then the composite scores obtained from the random classifications of unpredictability items into either stochasticity or volatility should be *more* correlated than when the scores result from our theory-driven classification. This intuition was confirmed by a permutation analysis, where we tested all possible ways of splitting our 7 unpredictability indicators into two composite scores (with 4 and 3 indicators in each composite). The correlation between the original, theory-driven Stochasticity and Volatility composites was indeed lower than any other possible combination (Supplementary Fig. S4, original r = 0.086, mean r of mixed composites = 0.192, t_(15)_ = 7.711, p < 0.001). Importantly, this was also the case for the correlation between Deprivation and Threat (Supplementary Fig. S5, original r = 0.208, mean r of mixed composites = 0.422, t_(8)_ = 6.724, p < 0.001), indicating that they are also relatively independent, as previous theory and empirical work suggests^6,7,31^.

### Pre-registered analyses

To test our main hypothesis that early life Stochasticity and Volatility contribute to the development of psychopathology during childhood and adolescence we carried out a set of pre-registered linear regressions. For each of our 4 outcome variables (internalising and externalising behaviours at ages 9 and 15), we fit and compared 4 models: a *Baseline* model with only control variables; an *Overall unpredictability* model, which additionally contained a main effect of an unpredictability composite that did not separate stochasticity and volatility; a *Stochasticity, volatility main effects* model, which contained the main effects of stochasticity and volatility, and a *Stochasticity, volatility interaction* model that further contained an interaction between stochasticity and volatility. We used the AIC for model selection, but BIC values are also reported (Table 2). We expected that in all cases, one of the models that contain separate terms for Stochasticity and Volatility would provide the best fit.

**Table 2:** Mean AIC, BIC and pooled R^2^ across imputations of the pre-registered linear models for the 4 outcomes.

	Internalising age 9			Externalising age 9			Internalising age 15			Externalising age 15		
	Mean AIC	Mean BIC	Pooled R^2^	Mean AIC	Mean BIC	Pooled R^2^	Mean AIC	Mean BIC	Pooled R^2^	Mean AIC	Mean BIC	Pooled R^2^
Model 1: Baseline	4382.706	**4420.532**	0.162	4192.254	**4230.080**	0.254	6038.523	**6078.454**	0.114	5671.898	**5711.828**	0.249
Model 2: Overall unpredictability	**4382.286**	4425.515	0.163	4193.855	4237.084	0.254	6033.807	6079.442	0.117	5668.410	5714.045	0.251
Model 3: Stochasticity, volatility main effects	4384.128	4432.761	0.163	4193.359	4241.992	0.255	**6029.149**	6080.488	0.119	**5665.135**	5716.475	0.253
Model 4: Stochasticity, volatility interaction	4386.050	4440.087	0.163	**4190.701**	4244.738	**0.257**	6030.955	6087.999	0.120	5666.862	5723.906	0.253

The lowest values, indicating best fitting models are highlighted in bold.

#### Internalising behaviours at age 9

The best fitting model was Model 2 (*Overall unpredictability,* see Table 2), with an AIC of 4382.286 (all other AICs ≥ 4382.706) and a percentage of explained variance (pooled R^2^) of 0.163 (see Supplementary Table S2 for the full regression table). In this model, internalising behaviours at age 5 (β = 0.387, 95% CI [0.341, 0.433], p < 0.001) were positively associated with internalising behaviours at age 9. No other terms were statistically significant.

#### Externalising behaviours at age 9

**Figure 1:**
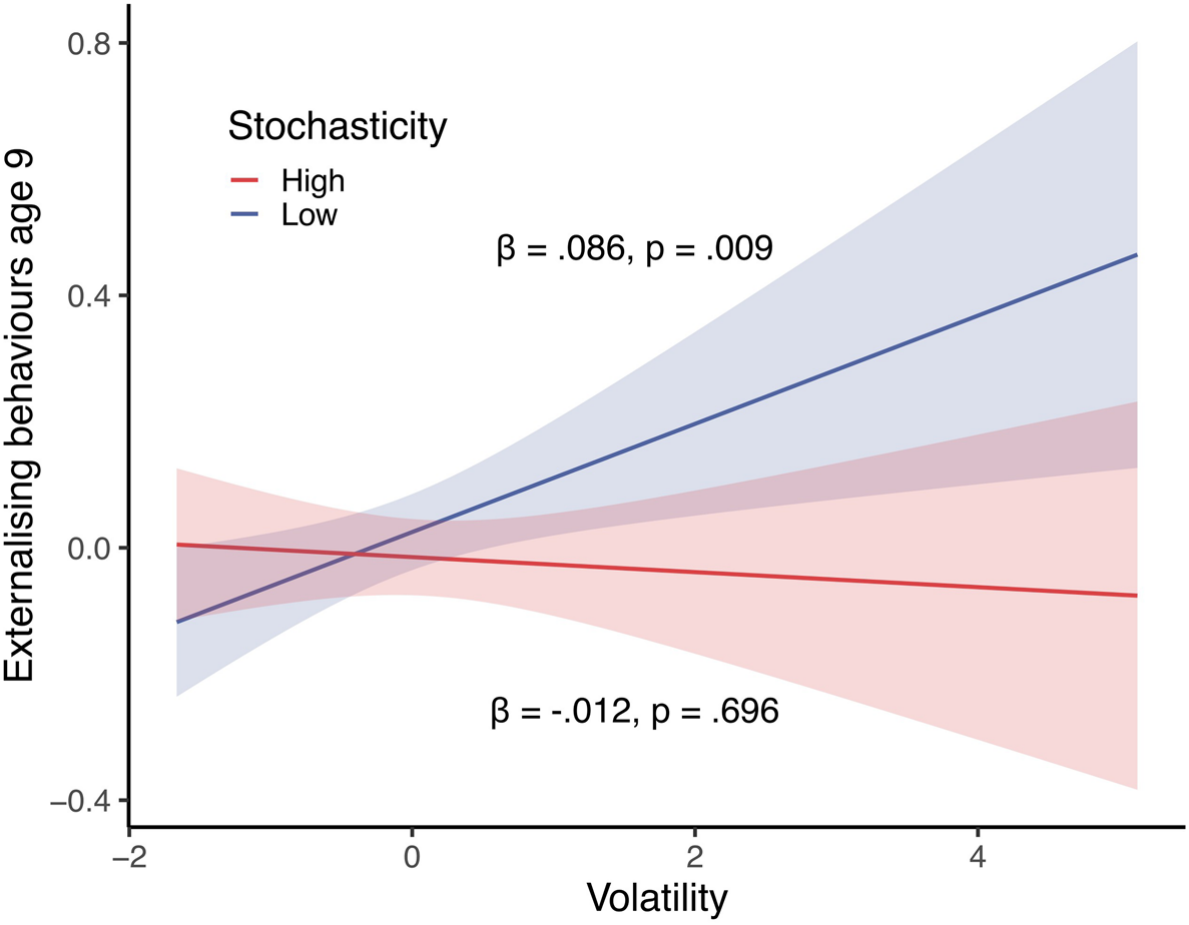
Interactive effect of volatility and stochasticity on externalising behaviours at age 9. Marginal effect of volatility on age 9 externalising behaviours in the best fitting linear model, conditioned on low (1SD below the mean) and high (1SD above the mean) values of stochasticity. The scale of externalising behaviours does not start at 0 due to standardization.

The best fitting model was Model 4 (*Stochasticity, volatility interaction*, see Table 2), with an AIC of 4190.701 (all other AICs ≥ 4192.254) and a percentage of explained variance (pooled R^2^) of 0.257 (Supplementary Table S3). In this model, Deprivation (β = 0.066, 95% CI [0.019, 0.114], p = 0.006) and Threat (β = 0.054, 95% CI [0.003, 0.104], p = 0.037) were positively associated with externalising behaviours at age 9. Interestingly, Volatility also interacted significantly with Stochasticity (β =  − 0.046, 95% CI [− 0.091, − 0.002], p = 0.041). A simple slopes analysis revealed that the positive association between Volatility and externalising behaviours was only apparent for lower levels of Stochasticity (β = 0.086, p = 0.009), and turned non-significant for higher levels (β =  − 0.012, p = 0.696) (Fig. 1). In other words, high levels in externalizing behaviours were not due to an additive effect of both forms of early life unpredictability. Rather, they seemed better explained by the ratio between the two, with Stochasticity potentially buffering against the effect of Volatility. Sensitivity analyses revealed that the significance of this effect was not robust to alternative composite specifications, although the direction and size of the effect was consistent (see Supplementary Fig. S2). In addition, externalising behaviours at age 5 (β = 0.438, 95% CI [0.392, 0.485], p < 0.001) and being male (β = 0.128, 95% CI [0.044, 0.213], p = 0.003) were also associated with increased externalising behaviours. No other terms were statistically significant.

#### Internalising behaviours at age 15

The best fitting model was Model 3 (*Stochasticity, volatility main effects*, see Table 2), with an AIC of 6029.149 (all other AICs ≥ 6030.955) and a percentage of explained variance (pooled R^2^) of 0.119 (Supplementary Table S4). In this model, volatility (β = 0.074, 95% CI [0.033, 0.116], p < 0.001) was positively associated with internalising behaviours at age 15. Sensitivity analyses revealed that this effect was robust to all alternative composite specifications (see Supplementary Fig. S2). In addition, internalising behaviours at age 9 (β = 0.319, 95% CI   [0.279, 0.358], p < 0.001) and being female (β = 0.136, 95% CI [0.057, 0.215], p < 0.001) were also positively associated with internalising symptoms at age 15. No other terms were statistically significant.

#### Externalising behaviours at age 15

The best fitting model was Model 3 (*Stochasticity, volatility main effects*, see Table 2), with an AIC of 5665.135 (all other AICs ≥ 5666.862) and a percentage of explained variance (pooled R^2^) of 0.253 (Supplementary Table S5). In this model, Deprivation (β = 0.060, 95% CI [0.016, 0.104], p = 0.007), Threat (β = 0.114, 95% CI [0.071, 0.157], p < 0.001) and Volatility (β = 0.061, 95% CI [0.023, 0.099], p = 0.002) were positively associated with the severity of externalising behaviours at age 15. Sensitivity analyses revealed that the effect of Volatility was robust to alternative composite specifications except when the Separations (β = 0.037, p = 0.068) indicator was dropped (see Supplementary Fig. S2). In addition, having grown up in a lower family SES (β =  − 0.055, 95% CI [− 0.093, − 0.017], p = 0.004) was associated with increased externalising problems. Individuals with higher levels of externalising behaviours at age 9 also expressed more externalising behaviours at this age (β = 0.416, 95% CI [0.378, 0.455], p < 0.001). No other terms were statistically significant.

### Unregistered analyses

In an unregistered follow-up analysis of our main results, we wished to test whether the uncovered effects of Volatility on adolescent problem behaviours are mediated by reproductive effort, an important component of human life history strategies^47^. To this end, we constructed a latent mediator model, in which the early life unpredictability composites had both a direct effect on age 15 problem behaviours, and indirect effects, mediated by a latent variable, aimed at capturing reproductive effort.

**Figure 2:**
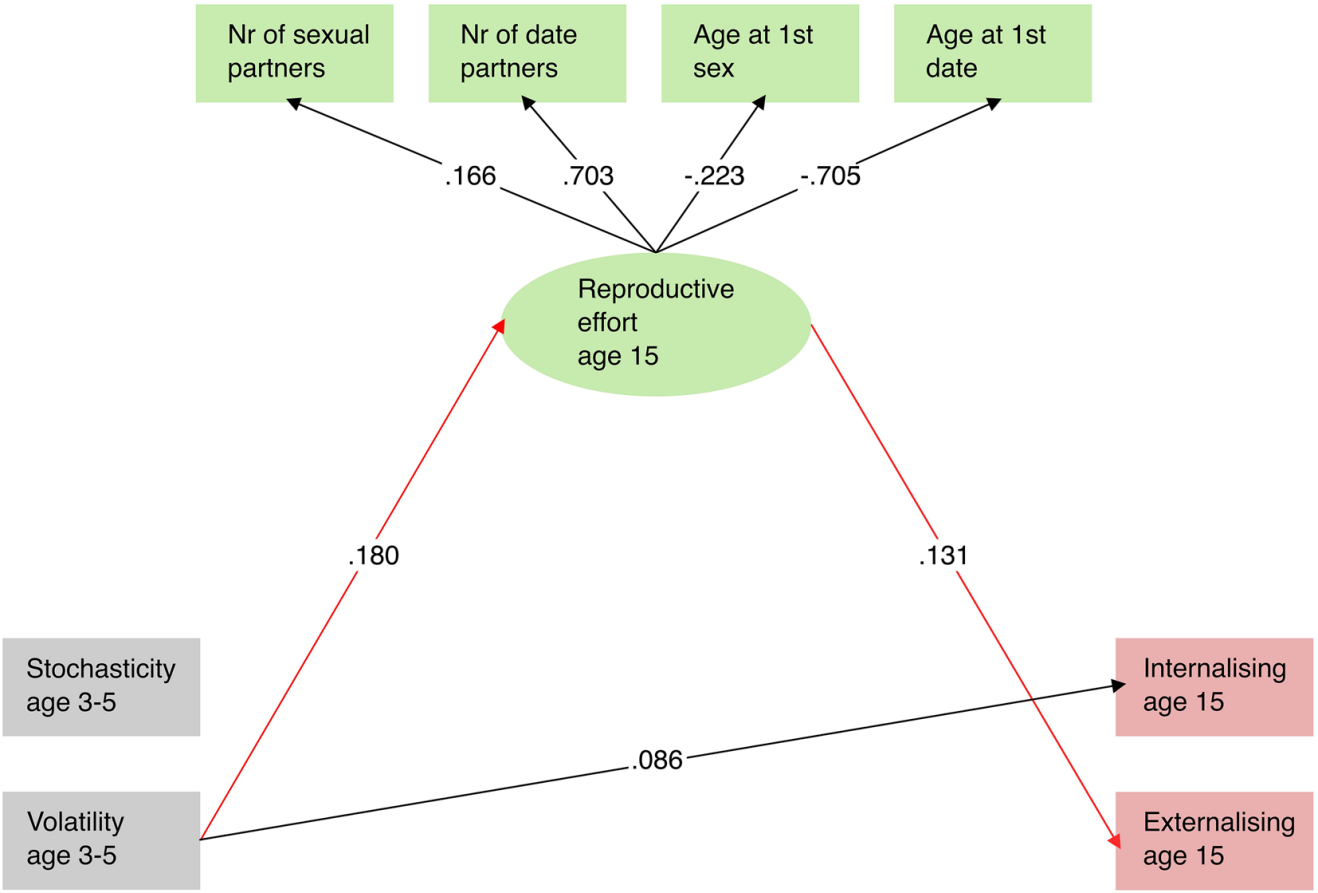
Simplified representation of the latent mediator structural equation model. Structures and standardised parameters. Ellipses represent latent variables, rectangles represent their indicators, directly observed measures. Significant paths are represented by arrows. The regression paths representing the statistically significant indirect effect of volatility on externalising behaviours are highlighted in red.

This model provided an excellent fit to the data (CFI = 0.919, RMSEA = 0.047 (90% CI [0.038, 0.055]), SRMR = 0.073). As expected, in the measurement model of the reproductive effort latent variable, the Number of sexual partners (β = 0.162, p < 0.001) and Number of date partners (β = 0.699, p < 0.001) indicators correlated positively with the latent, whereas the Age at 1st sexual intercourse (β =  − 0.219, p < 0.001) and Age at 1st date (β =  − 0.710, p < 0.001) indicators correlated negatively (Fig. 2, Supplementary Table S10). Thus, participants with earlier ages at 1st sexual intercourse and dating also tended to have more sexual and dating partners overall. Higher values on this latent thus correspond to a relatively higher reproductive effort. The structural model further indicated that Volatility (β = 0.183, p < 0.001) was significantly and positively related to reproductive effort, meaning that, experiencing higher levels of early life Volatility is associated with increased reproductive effort during adolescence. Increased reproductive effort was also positively related to externalising (β = 0.131, p < 0.001), but not internalising behaviours. Additionally, Volatility had a direct effect on internalising (β = 0.086, p = 0.001), but not externalising behaviours. The mediation analyses revealed that Volatility had a significant total effect (β = 0.063, p = 0.013) and a significant indirect effect (β = 0.024, p = 0.001) mediated by reproductive effort on externalising behaviours and a significant total effect on internalising behaviours (β = 0.082, p = 0.001). No other direct, total or indirect effect was statistically significant.

## Discussion

### Distinct effects of early life stochasticity and volatility revealed by the pre-registered analyses

In this study, we modelled the emergence of childhood and adolescent symptoms of psychopathology as a consequence of multiple dimensions of early life environments. Specifically, we investigated the environmental correlates of internalising and externalising behaviours at childhood (age 9) and adolescence (age 15) by focusing on the dimensions of Deprivation, Threat, and Unpredictability. Regarding the effects of Deprivation and Threat; our results confirm previous findings in showing a strong effect of Threat on the severity of externalising problems, with a weaker effect of Deprivation, although we failed to replicate earlier work showing effects on internalising behaviours^6,31,54^. The novelty of our study is our distinction between two different timescales of unpredictability, short timescale, unpredictability (Stochasticity) on the scale of days and weeks, and long timescale unpredictability (Volatility) on the scale of months and years. We expected this novel conceptual distinction to have an empirical utility in predicting these various psychopathological outcomes over and above the effects of Deprivation and Threat.

This hypothesis was partially confirmed, as models including separate stochasticity and volatility variables proved more efficient in fitting the severity of externalising behaviours at both ages and internalising behaviours at age 15. Our results imply that, children exposed to volatility factors such as parental transitions or moves might be more likely to develop high levels of especially externalising behaviours, and especially during adolescence, whereas exposure to stochasticity factors such as home chaos and lack of family routines might have more subtle ways of influencing development, that we were unable to detect in this work.

### Adolescent reproductive effort as a potential mediator revealed by the unregistered analysis

In an unregistered follow-up analysis of our main results, we wished to test whether the uncovered effects of Volatility on adolescent problem behaviours are mediated by reproductive effort, an important component of human life history strategies^47^. The development of an accelerated life history strategy—i.e., a strategy that provides reproductive benefits at an earlier age—is indeed expected to go hand in hand with the emergence of psychological traits that facilitate its achievement, such like sensation-seeking, impulsivity, disinhibition. These traits are known to be involved in short-term reproductive goals like lower mate selectivity, sexual promiscuity, intrasexual competition for mates and status, or lower parenting effort, and are the core components of externalizing behaviours^55–57^. We reasoned that if Volatility impacts problem behaviours to a greater degree due to its relatively larger influence on predictive plasticity, then its effect should be mediated by the adoption of accelerated life history strategies. We expected higher Volatility to lead to accelerated life courses, indexed by higher and earlier reproductive effort. To this end, we constructed a latent mediator model, in which the early life unpredictability composites had both a direct effect on age 15 problem behaviours, and indirect effects, mediated by a latent variable, aimed at capturing reproductive effort. The results of this model lend support to this idea, as volatility was the only adversity dimension with an indirect effect on psychopathology that was mediated by reproductive effort, an important life history trait.

### Unpredictability as an adaptive challenge

Unpredictability in the environment poses an adaptive challenge that can be mitigated by multiple types of plasticity, such as predictive plasticity or bet-hedging^8,12,58^. The relative importance of these processes is determined by a number of factors, including the timescale of variability^19^. Variability that is too quick relative to the organisms capacity for developing a matching phenotype should not lead to significant predictive plasticity^16,17,59,60^. Such plasticity will be favoured when fluctuations in environmental conditions are frequent enough to pose an adaptive challenge but still infrequent enough so that developing a matching phenotype is possible. Translating this reasoning into human development, short timescale unpredictability on the order of days and weeks is unlikely to lead to forward looking predictive adaptive responses, like commitment to a ‘fast’ life history. However, short timescale unpredictability might still lead to developmental responses that are adaptive in the present (Level 1 adaptations in the terminology of the recent theoretical model of Ellis et al.^10^), such as increased vigilance and threat detection^61–63^ and certain attachment styles^64,65^, which we were not able to study. Thus, a possible explanation of the fact that unlike stochasticity, volatility is specifically linked with high levels of externalising behaviours, is that these two timescales of unpredictability lead to adaptive responses at different levels, with different consequences for mental and physical health.

Although previous studies have not explicitly investigated the specific associations between different timescales of unpredictability and internalising and externalising dimensions, some preliminary results do suggest a relatively stronger relationship between stochasticity and internalising behaviours, and volatility and externalising behaviours. In the study of Doom et al.^12^, an early unpredictability metric, comprising volatility, was associated with externalising behaviours. Whereas in the study of Glynn et al.^66^, maternal mood entropy, a measure more closely indicating stochasticity, was associated with child internalising problems at multiple ages from childhood to adolescence. In another study, Li et al.^67^ created a composite early life unpredictability measure, reflecting both stochastic and volatile unpredictability, but with a predominant longer term component. In their results, the authors found that this composite was associated with both externalising and internalising behaviours. However, the effect on externalising behaviours was stronger. The study of Chang et al.^68^ included measurements of both negative life events (a volatility factor) and home chaos (a stochasticity factor), although in their structural equation model they were all indicators of a unique general adversity latent construct. Nevertheless, at the level of pairwise correlations, negative life events were significantly correlated with increases in externalising symptoms, whereas home chaos was significantly correlated with increases in internalising symptoms during the COVID-19 pandemic. Finally, Li and Belsky^69^ investigated the effects of income harshness (defined by average income-to-needs ratio) and income unpredictability (defined as variability in income-to-needs across 4 time points). Under our view, such income unpredictability should be interpreted as volatility, as it likely reflects change points in the financial and working conditions of the parents. In accordance, the authors found that whereas income harshness was related both directly and indirectly to both externalising and internalising behaviours, income unpredictability was only related to externalising behaviours.

The surprising pattern of interaction between stochasticity and volatility in predicting childhood externalising behaviours is also noteworthy. Externalising behaviours at age 9 were highest among children who experienced high volatility but low stochasticity. As a reminder, our reasoning is that the increased externalising behaviours following volatility reflect predictive adaptive plasticity to harsh environmental conditions. If correct, then it is possible that the overlap of longer term with shorter term fluctuations signals an environment that is changing too fast for predictive plasticity to occur. We indeed noted how such plasticity might be mostly observed at intermediate rates of environmental change. It might be that the presence of shorter term fluctuations makes the longer term fluctuations noisier. The lower signal-to-noise ratio of the environment might thus push the unpredictability timescale into the ‘too short’ ranges, which might thus buffer children against the expected effects of volatile unpredictability on impulsive decision-making. This also echoes the opposing effects of stochastic and volatile uncertainty on learning rates in the reinforcement learning literature^27,28^. According to optimal learning models, stochastic uncertainty should decrease learning rates and lead to longer integration windows, whereas volatile uncertainty should increase learning rates and lead to shorter integration windows. If we interpret developmental plasticity as analogous to learning rates, then this is exactly what our pattern of interaction suggests. An alternative possibility is that the buffering effect played by stochasticity over volatility stems from the way unpredictability relates to parenting styles. For example, less stochastic bedtime routines could be due to more authoritative parenting styles, which have been related to socio-emotional adjustment and child mental health^70^.

### Limitations

There are a number of limitations of our study that need to be mentioned and addressed in future work. First and foremost, the models we tested here are constrained by the nature of the data, which led us to operationalise our two unpredictability variables as dimensions that might appear orthogonal to the dimensions of threat and deprivation. Nevertheless, we are fully aware that it would be more realistic to consider factors of threat and deprivation to be themselves subject to a certain degree of unpredictability. In addition, the indicators themselves probably lack precision. For example, the indicators of deprivation that we have at hand—i.e., frequency of activities where the mother engage with the child, frequency of parent–child interactions, counting toys and books at the disposal of the child—do not take into account the quality and duration of the interactions between the parents and the child, or the possibility that the child can optimally benefit from a number of toys or books that is not too excessive. In a similar vein, our indicators of stochasticity and volatility also differ in features other than timescale, for example in severity. Our analytic approach of constructing dimension specific composite scores mitigates this concern to a degree, and allowed us to approximate children’s environments on the dimensions that we wished to focus on. The robustness of the volatility effects to alternative composite score specifications (Supplementary Fig. S3) also suggests this to be the case. This approach is also in line with the assumption of the recent dimensional model of adversity, that proposes that children integrate multiple different experiences into overall estimates of environmental states^5,10^. These limitations nevertheless raise the importance of developing new tools allowing for a more precise quantification of adversity factors in general, including the timescale at which they fluctuate. Secondly, we attribute the effects we found to developmental processes, initiated at early ages by environmental factors. However, we lacked information about the status of these same environmental factors at the ages that our outcome variables were measured. Thus, if early life environmental factors were strongly correlated between early life, childhood and adolescence, they could also explain the effects. We tried to control for current environmental adversity by including the current SES in our models. However, future studies should ideally measure the same environmental factors at both past and current timepoints. Thirdly, we did not have any subjective ratings from the participating children about the severity and impact of the indicators. This is an important limitation, as there is reason to believe that perceptions of adverse events will have a stronger psychological and physiological impact, than the objective events themselves^71–75^. Moreover, having such data would also help to make sure that the distinct effects of stochasticity and volatility we observed are not due to differences in the accuracy of reporting or biases in retrospective recall between indicators. Therefore, future research should, if possible, also include subjective ratings of the impact and perception of adverse experiences.

We also note that effect sizes were rather low in all models, suggesting that: (i) the indicators we used as proxies of adversity dimensions are relatively poor, or (ii) the dimensions we considered explain only a fraction of the variance in childhood and adolescent psychopathology. We believe the likely answer is that a combination of both played a role (we already discussed the imperfect nature of our proxies above). It is also clear that there are sources of inter-individual variability in psychopathology, other than the adversity dimensions we considered here, for example other adversity factors that were not included in our composites (e.g., traumatic experiences, minority status, and other factors that cut across the dimensional models we employed)^75^, or genetics^76^. Relatedly, while our model selection criterion (the AIC) already penalises model complexity, it must be highlighted that differences between our models corresponded to very small differences in explained variance. Moreover, the BIC always favoured the simplest, Baseline model, further highlighting the marginal nature of the differences. This, along with the abovementioned shortcomings of our operationalisation of adversity dimensions raises the possibility that our findings do not translate into meaningful effect sizes or generalise to other samples. Clearly, much more research, and converging evidence from other operationalisations and modelling approaches is necessary before a clear interpretation of stochasticity and volatility as being associated with different adaptive developmental responses is warranted.

## Conclusion

To conclude, we have provided preliminary evidence for the utility of distinguishing short timescale, stochastic from long timescale, volatile unpredictability in developmental psychopathology research. Such a distinction helps explain individual differences in the emergence of behaviours than accompany different forms of psychopathology in childhood and adolescence. Short timescale, day-to-day unpredictability proved relatively unrelated to both internalizing and externalizing behaviours, whereas longer timescale unpredictability was associated with both externalising, and to smaller extent internalising behaviours. Our results also shed light on an important trade-off between short and long timescale term unpredictability: externalising behaviours were the highest when long timescale unpredictability was not coupled with short timescale unpredictability. We have interpreted these findings in terms of evolutionary-developmental and reinforcement learning models. Overall, we believe that this study adds nuance to current theories of how unpredictability shapes psychological and physiological development and sets the stage for future research.

### Supplementary Information

Supplementary Information.

## Data Availability

All pre-processed data and code necessary to reproduce all results in the paper are available at the OSF framework (https://osf.io/e98px/). The raw data that support the findings of this study are similarly available after the appropriate steps from the Fragile Families Child and Wellbeing Study website (https://fragilefamilies.princeton.edu/documentation).
