# Supplementary material for: Specifying the timescale of early life unpredictability helps explain the development of internalising and externalising behaviours
Source: Sci Rep. 2024 Feb 12;14:3563. doi: 10.1038/s41598-024-54093-x (PMC10861493; doi:10.1038/s41598-024-54093-x)
Supplement: Supplementary file 1 — Supplementary Information. [file 41598_2024_54093_MOESM1_ESM.docx]

**Specifying the timescale of early life unpredictability helps explain the development of internalising and externalising behaviours**

**Supplementary material**

| **Composite** | **Indicator** | **% missing data** |
| --- | --- | --- |
| Deprivation | Activities age 3 | 2.37 |
|  | Activities age 5 | 1.69 |
|  | Toys and books age 3 | 15.34 |
|  | Toys and books age 5 | 21.66 |
|  | Parent-child interactions age 3 | 44.47 |
|  | Parent-child interactions age 5 | 42.42 |
| Threat | Psychological aggression age 3 | 15.52 |
|  | Psychological aggression age 5 | 20.26 |
|  | Physical aggression age 3 | 16.13 |
|  | Physical aggression age 5 | 20.40 |
|  | Community violence age 3 | 15.12 |
|  | Community violence age 5 | 20.08 |
| ST unpredictability | Arrangements age 3 | 0.90 |
|  | Arrangements age 5 | 16.16 |
|  | Regular bedtime age 3 | 15.23 |
|  | Regular bedtime age 5 | 20.55 |
|  | Bedtime routine age 3 | 15.23 |
|  | Bedtime routine age 5 | 19.76 |
| LT unpredictability | Separations age 3 | 1.00 |
|  | Separations age 5 | 1.36 |
|  | Moves age 3 | 0.04 |
|  | Moves age 5 | 0.18 |
|  | Jobs age 3 | 43.25 |
|  | Jobs age 5 | 43.86 |
|  | Maternal depression change age 3 | 0.11* |
|  | Maternal depression change age 5 | 0.25* |
| - | Internalising behaviours age 5 | 26.01* |
|  | Internalising behaviours age 9 | 13.69* |
|  | Internalising behaviours age 15 | 1.83* |
|  | Externalising behaviours age 5 | 26.54* |
|  | Externalising behaviours age 9 | 12.07* |
|  | Externalising behaviours age 15 | 1.94* |
|  | SES age 9 | 0.90 |
|  | SES age 15 | 0.18 |
|  | Age at 1^st^ sexual intercourse | 18.43* |
|  | Age at 1^st^ date | 5.42* |
|  | Nr of sexual partners | 5.17* |
|  | Nr of date partners | 8.23* |

**Supplementary Table S1.** **Fraction of missing data for each indicator.**

* Missing cases in these variables were not imputed.

| Age 9 Internalising | β | SE | 95% CI | t | df | p | VIF |
| --- | --- | --- | --- | --- | --- | --- | --- |
| Intercept | -.010 | 0.032 | [-.072 .052] | -0.323 | 1632.702 | .747 |  |
| Depriv | -.010 | 0.026 | [-.061 .041] | -0.379 | 548.542 | .705 | 1.20 |
| Threat | .034 | 0.026 | [-.017 .085] | 1.308 | 765.448 | .191 | 1.21 |
| Unpredictability | .036 | 0.024 | [-.011 .084] | 1.493 | 1433.108 | .136 | 1.12 |
| SES | -.016 | 0.024 | [-.063 .031] | -0.652 | 1610.837 | .515 | 1.14 |
| SexFemale | .024 | 0.045 | [-.065 .113] | 0.522 | 1632.234 | .602 | 1.02 |
| Age 5 internalising | .387 | 0.024 | [.341 .433] | 16.400 | 1621.243 | < .001 | 1.12 |

**Supplementary Table S2.** **Results of the linear regression predicting Age 9 internalising behaviours.** All variables were standardized before entering into the regression, therefore coefficients are interpretable as standardized betas. Results are pooled estimates across 20 imputed datasets. Variance inflation factors, based on the unimputed data are also reported.

| Age 9 Externalising | β | SE | 95% CI | t | df | p | VIF |
| --- | --- | --- | --- | --- | --- | --- | --- |
| Intercept | .068 | 0.030 | [.010 .127] | 2.280 | 1627.989 | .023 |  |
| Depriv | .066 | 0.024 | [.019 .114] | 2.747 | 746.562 | .006 | 1.19 |
| Threat | .054 | 0.026 | [.003 .104] | 2.087 | 834.248 | .037 | 1.35 |
| Stoch | -.017 | 0.023 | [-.064 .029] | -0.743 | 850.738 | .457 | 1.06 |
| Volat | .033 | 0.023 | [-.011 .077] | 1.481 | 1423.748 | .139 | 1.10 |
| Stoch x  Volat | -.046 | 0.023 | [-.091 -.002] | -2.051 | 1083.521 | .041 | 1.03 |
| SES | -.035 | 0.023 | [-.079 .010] | -1.538 | 1614.459 | .124 | 1.15 |
| SexFemale | -.128 | 0.043 | [-.213 -.044] | -2.990 | 1628.220 | .003 | 1.03 |
| Age 5 externalising | .438 | 0.024 | [.392 .485] | 18.317 | 1568.638 | < .001 | 1.27 |

**Supplementary Table S3.** **Results of the linear regression predicting Age 9 externalising behaviours.** All variables were standardized before entering into the regression, therefore coefficients are interpretable as standardized betas. Results are pooled estimates across 20 imputed datasets. Variance inflation factors, based on the unimputed data are also reported.

| Age 15  Internalising | β | SE | 95% CI | t | df | p | VIF |
| --- | --- | --- | --- | --- | --- | --- | --- |
| Intercept | -.068 | 0.028 | [-.122 -.013] | -2.419 | 2207.076 | .016 |  |
| Depriv | .009 | 0.022 | [-.035 .053] | 0.408 | 983.176 | .684 | 1.19 |
| Threat | .033 | 0.023 | [-.012 .077] | 1.444 | 1225.266 | .149 | 1.22 |
| Stoch | -.008 | 0.022 | [-.051 .035] | -0.360 | 915.648 | .719 | 1.06 |
| Volat | .074 | 0.021 | [.033 .116] | 3.515 | 1588.816 | < .001 | 1.07 |
| SES | .012 | 0.021 | [-.029 .053] | 0.593 | 2172.062 | .553 | 1.13 |
| SexFemale | .136 | 0.040 | [.057 .215] | 3.389 | 2205.917 | < .001 | 1.03 |
| Age 9 internalising | .319 | 0.020 | [.279 .358] | 15.776 | 2202.112 | < .001 | 1.04 |

**Supplementary Table S4.** **Results of the linear regression predicting Age 15 internalising behaviours.** All variables were standardized before entering into the regression, therefore coefficients are interpretable as standardized betas. Results are pooled estimates across 20 imputed datasets. Variance inflation factors, based on the unimputed data are also reported.

| Age 15  Externalising | β | SE | 95% CI | t | df | p | VIF |
| --- | --- | --- | --- | --- | --- | --- | --- |
| Intercept | -.007 | 0.026 | [-.057 .044] | -0.260 | 2202.113 | .795 |  |
| Depriv | .060 | 0.022 | [.016 .104] | 2.701 | 329.546 | .007 | 1.19 |
| Threat | .114 | 0.022 | [.071 .157] | 5.218 | 711.878 | < .001 | 1.27 |
| Stoch | -.007 | 0.021 | [-.048 .035] | -0.309 | 429.678 | .758 | 1.06 |
| Volat | .061 | 0.020 | [.023 .099] | 3.117 | 1420.755 | .002 | 1.07 |
| SES | -.055 | 0.019 | [-.093 -.017] | -2.865 | 2155.441 | .004 | 1.12 |
| SexFemale | .017 | 0.037 | [-.056 .090] | 0.465 | 2199.341 | .642 | 1.03 |
| Age 9 externalising | .416 | 0.020 | [.378 .455] | 21.354 | 2155.705 | < .001 | 1.12 |

**Supplementary Table S5.** **Results of the linear regression predicting Age 15 externalising behaviours.** All variables were standardized before entering into the regression, therefore coefficients are interpretable as standardized betas. Results are pooled estimates across 20 imputed datasets. Variance inflation factors, based on the unimputed data are also reported.

| **Variable** |  | **Internalising age 9**  **Not missing** | **Internalising age 9**  **Missing** | **p** | **p Benjamini-Hochberg** |
| --- | --- | --- | --- | --- | --- |
| Sex | Male | 1242 (86.9) | 188 (13.1) | .427 | .728 |
|  | Female | 1161 (85.7) | 193 (14.3) |  |  |
| SES age 9 | Mean (SD) | 2.1 (2.3) | 2.1 (2.5) | .812 | .906 |
| SES age 15 | Mean (SD) | 2.4 (2.6) | 2.4 (2.5) | .648 | .859 |
| Activities age 3 | Mean (SD) | -0.0 (1.0) | 0.1 (1.0) | .262 | .691 |
| Activities age 5 | Mean (SD) | -0.0 (1.0) | 0.2 (1.0) | .002 | .058 |
| Toys and books age 3 | Mean (SD) | -0.1 (1.0) | 0.0 (1.0) | .190 | .612 |
| Toys and books age 5 | Mean (SD) | -0.0 (1.0) | 0.0 (1.0) | .236 | .684 |
| Interactions age 3 | Mean (SD) | -0.1 (1.0) | 0.1 (1.0) | .015 | .218 |
| Interactions age 5 | Mean (SD) | -0.0 (1.0) | 0.1 (1.0) | .308 | .713 |
| Psychological aggression age 3 | Mean (SD) | 8.2 (4.8) | 8.3 (4.7) | .605 | .859 |
| Psychological aggression age 5 | Mean (SD) | 8.8 (5.0) | 8.3 (5.2) | .090 | .522 |
| Physical aggression age 3 | Mean (SD) | 6.5 (5.1) | 6.0 (4.9) | .069 | .522 |
| Physical aggression age 5 | Mean (SD) | 5.7 (4.9) | 5.6 (5.3) | .897 | .929 |
| Community violence age 3 | Mean (SD) | 1.2 (2.3) | 1.2 (2.2) | .772 | .906 |
| Community violence age 5 | Mean (SD) | 1.1 (2.4) | 0.9 (1.9) | .169 | .612 |
| Arrangements age 3 | Mean (SD) | 0.7 (0.6) | 0.7 (0.7) | .968 | .968 |
| Arrangements age 5 | Mean (SD) | 0.1 (0.4) | 0.1 (0.4) | .464 | .748 |
| Regular bedtime age 3 | Mean (SD) | 2.4 (2.0) | 2.4 (2.0) | .703 | .886 |
| Regular bedtime age 5 | Mean (SD) | 2.0 (1.5) | 1.8 (1.5) | .183 | .612 |
| Bedtime routine age 3 | Mean (SD) | 2.2 (1.9) | 2.1 (1.9) | .352 | .713 |
| Bedtime routine age 5 | Mean (SD) | 2.5 (2.0) | 2.3 (1.9) | .353 | .713 |
| Maternal depression change age 3 | No | 1905 (86.5) | 298 (13.5) | .652 | .859 |
|  | Yes | 495 (85.6) | 83 (14.4) |  |  |
| Maternal depression change age 5 | No | 1901 (86.0) | 309 (14.0) | .404 | .728 |
|  | Yes | 496 (87.5) | 71 (12.5) |  |  |
| Separations age 3 | Mean (SD) | 0.2 (0.6) | 0.2 (0.6) | .369 | .713 |
| Separations age 5 | Mean (SD) | 0.3 (0.7) | 0.3 (0.7) | .889 | .929 |
| Moves age 3 | Mean (SD) | 0.6 (0.8) | 0.7 (0.9) | .135 | .612 |
| Moves age 5 | Mean (SD) | 0.7 (0.8) | 0.7 (0.8) | .811 | .906 |
| Jobs age 3 | Mean (SD) | 1.5 (1.0) | 1.5 (1.0) | .492 | .751 |
| Jobs age 5 | Mean (SD) | 1.3 (0.9) | 1.2 (0.9) | .080 | .522 |

**Supplementary Table S6.** **Missing data analysis for the Internalising behaviours age 9 outcome variable.** Comparisons for continuous data use a Kruskal Wallis and for discrete data a chi-squared test. No difference survives correction for multiple comparisons.

| **Variable** |  | **Internalising age 15**  **Not missing** | **Internalising age 15**  **Missing** | **p** | **p Benjamini-Hochberg** |
| --- | --- | --- | --- | --- | --- |
| Sex | Male | 1397 (97.7) | 33 (2.3) | .075 | .725 |
|  | Female | 1336 (98.7) | 18 (1.3) |  |  |
| SES age 9 | Mean (SD) | 2.1 (2.4) | 1.2 (1.0) | .010 | .290 |
| SES age 15 | Mean (SD) | 2.4 (2.6) | 1.6 (1.3) | .034 | .493 |
| Activities age 3 | Mean (SD) | -0.0 (1.0) | 0.1 (0.9) | .657 | .891 |
| Activities age 5 | Mean (SD) | 0.0 (1.0) | 0.2 (1.0) | .271 | .823 |
| Toys and books age 3 | Mean (SD) | -0.1 (1.0) | -0.0 (1.0) | .780 | .891 |
| Toys and books age 5 | Mean (SD) | -0.0 (1.0) | -0.1 (1.0) | .837 | .899 |
| Interactions age 3 | Mean (SD) | -0.0 (1.0) | -0.1 (0.9) | .765 | .891 |
| Interactions age 5 | Mean (SD) | -0.0 (1.0) | 0.1 (1.1) | .623 | .891 |
| Psychological aggression age 3 | Mean (SD) | 8.2 (4.8) | 9.1 (4.5) | .171 | .823 |
| Psychological aggression age 5 | Mean (SD) | 8.8 (5.0) | 8.3 (5.2) | .540 | .891 |
| Physical aggression age 3 | Mean (SD) | 6.4 (5.0) | 6.8 (5.5) | .668 | .891 |
| Physical aggression age 5 | Mean (SD) | 5.7 (4.9) | 6.2 (4.9) | .497 | .891 |
| Community violence age 3 | Mean (SD) | 1.2 (2.3) | 1.3 (2.5) | .795 | .891 |
| Community violence age 5 | Mean (SD) | 1.1 (2.4) | 1.2 (2.3) | .674 | .891 |
| Arrangements age 3 | Mean (SD) | 0.7 (0.6) | 0.8 (0.8) | .505 | .891 |
| Arrangements age 5 | Mean (SD) | 0.1 (0.4) | 0.1 (0.4) | .794 | .891 |
| Regular bedtime age 3 | Mean (SD) | 2.4 (1.9) | 2.8 (2.2) | .213 | .823 |
| Regular bedtime age 5 | Mean (SD) | 2.0 (1.5) | 2.1 (1.8) | .544 | .891 |
| Bedtime routine age 3 | Mean (SD) | 2.2 (1.9) | 2.0 (1.8) | .559 | .891 |
| Bedtime routine age 5 | Mean (SD) | 2.5 (2.0) | 2.0 (1.9) | .189 | .823 |
| Maternal depression change age 3 | No | 2166 (98.3) | 37 (1.7) | .312 | .823 |
|  | Yes | 564 (97.6) | 14 (2.4) |  |  |
| Maternal depression change age 5 | No | 2170 (98.2) | 40 (1.8) | .999 | .999 |
|  | Yes | 557 (98.2) | 10 (1.8) |  |  |
| Separations age 3 | Mean (SD) | 0.2 (0.6) | 0.4 (0.8) | .147 | .823 |
| Separations age 5 | Mean (SD) | 0.3 (0.7) | 0.4 (0.6) | .295 | .823 |
| Moves age 3 | Mean (SD) | 0.6 (0.8) | 0.8 (0.9) | .246 | .823 |
| Moves age 5 | Mean (SD) | 0.7 (0.8) | 0.6 (0.8) | .799 | .891 |
| Jobs age 3 | Mean (SD) | 1.5 (1.0) | 1.5 (1.1) | .987 | .999 |
| Jobs age 5 | Mean (SD) | 1.3 (0.9) | 1.2 (0.8) | .420 | .891 |

**Supplementary Table S7.** **Missing data analysis for the Internalising behaviours age 15 outcome variable.** Comparisons for continuous data use a Kruskal Wallis and for discrete data a chi-squared test. No difference survives correction for multiple comparisons.

| **Variable** |  | **Externalising age 9**  **Not missing** | **Externalising age 9**  **Missing** | **p** | **p Benjamini-Hochberg** |
| --- | --- | --- | --- | --- | --- |
| Sex | Male | 1260 (88.1) | 170 (11.9) | .808 | .901 |
|  | Female | 1188 (87.7) | 166 (12.3) |  |  |
| SES age 9 | Mean (SD) | 2.1 (2.3) | 2.2 (2.5) | .380 | .648 |
| SES age 15 | Mean (SD) | 2.4 (2.6) | 2.6 (2.7) | .232 | .567 |
| Activities age 3 | Mean (SD) | -0.0 (1.0) | 0.1 (1.0) | .250 | .567 |
| Activities age 5 | Mean (SD) | -0.0 (1.0) | 0.1 (1.0) | .009 | .131 |
| Toys and books age 3 | Mean (SD) | -0.1 (1.0) | -0.0 (1.0) | .688 | .867 |
| Toys and books age 5 | Mean (SD) | -0.0 (1.0) | 0.0 (1.1) | .148 | .464 |
| Interactions age 3 | Mean (SD) | -0.1 (1.0) | 0.2 (1.1) | .007 | .131 |
| Interactions age 5 | Mean (SD) | -0.0 (1.0) | 0.2 (1.0) | .019 | .181 |
| Psychological aggression age 3 | Mean (SD) | 8.2 (4.8) | 8.0 (4.7) | .576 | .779 |
| Psychological aggression age 5 | Mean (SD) | 8.8 (5.0) | 8.6 (5.0) | .541 | .779 |
| Physical aggression age 3 | Mean (SD) | 6.5 (5.0) | 6.3 (5.1) | .591 | .779 |
| Physical aggression age 5 | Mean (SD) | 5.6 (4.9) | 6.0 (5.2) | .306 | .624 |
| Community violence age 3 | Mean (SD) | 1.2 (2.3) | 1.3 (2.6) | .254 | .567 |
| Community violence age 5 | Mean (SD) | 1.1 (2.4) | 1.1 (2.3) | .889 | .929 |
| Arrangements age 3 | Mean (SD) | 0.7 (0.7) | 0.7 (0.6) | .380 | .648 |
| Arrangements age 5 | Mean (SD) | 0.1 (0.4) | 0.1 (0.4) | .455 | .733 |
| Regular bedtime age 3 | Mean (SD) | 2.4 (2.0) | 2.4 (1.9) | .739 | .869 |
| Regular bedtime age 5 | Mean (SD) | 2.0 (1.5) | 1.9 (1.5) | .323 | .624 |
| Bedtime routine age 3 | Mean (SD) | 2.2 (1.9) | 2.2 (1.9) | .999 | .999 |
| Bedtime routine age 5 | Mean (SD) | 2.4 (2.0) | 2.6 (2.0) | .160 | .464 |
| Maternal depression change age 3 | No | 1953 (88.7) | 250 (11.3) | .025 | .181 |
|  | Yes | 492 (85.1) | 86 (14.9) |  |  |
| Maternal depression change age 5 | No | 1942 (87.9) | 268 (12.1) | .897 | .929 |
|  | Yes | 500 (88.2) | 67 (11.8) |  |  |
| Separations age 3 | Mean (SD) | 0.2 (0.6) | 0.3 (0.6) | .749 | .869 |
| Separations age 5 | Mean (SD) | 0.3 (0.7) | 0.4 (0.8) | .078 | .435 |
| Moves age 3 | Mean (SD) | 0.6 (0.8) | 0.7 (0.8) | .090 | .435 |
| Moves age 5 | Mean (SD) | 0.7 (0.8) | 0.7 (0.9) | .134 | .464 |
| Jobs age 3 | Mean (SD) | 1.5 (1.0) | 1.5 (1.0) | .487 | .743 |
| Jobs age 5 | Mean (SD) | 1.3 (0.9) | 1.2 (0.9) | .117 | .464 |

**Supplementary Table S8.** **Missing data analysis for the Externalising behaviours age 9 outcome variable.** Comparisons for continuous data use a Kruskal Wallis and for discrete data a chi-squared test. No difference survives correction for multiple comparisons.

| **Variable** |  | **Externalising age 15**  **Not missing** | **Externalising age 15**  **Missing** | **p** | **p Benjamini-Hochberg** |
| --- | --- | --- | --- | --- | --- |
| Sex | Male | 1401 (98.0) | 29 (2.0) | .834 | .864 |
|  | Female | 1329 (98.2) | 25 (1.8) |  |  |
| SES age 9 | Mean (SD) | 2.1 (2.4) | 1.4 (1.2) | .027 | .283 |
| SES age 15 | Mean (SD) | 2.4 (2.6) | 1.9 (1.5) | .104 | .377 |
| Activities age 3 | Mean (SD) | -0.0 (1.0) | 0.3 (1.1) | .039 | .283 |
| Activities age 5 | Mean (SD) | -0.0 (1.0) | 0.2 (1.0) | .283 | .746 |
| Toys and books age 3 | Mean (SD) | -0.1 (1.0) | 0.1 (0.9) | .191 | .615 |
| Toys and books age 5 | Mean (SD) | -0.0 (1.0) | 0.0 (1.0) | .598 | .864 |
| Interactions age 3 | Mean (SD) | -0.0 (1.0) | 0.0 (1.0) | .793 | .864 |
| Interactions age 5 | Mean (SD) | -0.0 (1.0) | -0.1 (0.9) | .676 | .864 |
| Psychological aggression age 3 | Mean (SD) | 8.1 (4.8) | 9.5 (5.1) | .055 | .315 |
| Psychological aggression age 5 | Mean (SD) | 8.7 (5.0) | 9.2 (4.7) | .553 | .864 |
| Physical aggression age 3 | Mean (SD) | 6.4 (5.1) | 8.0 (4.4) | .038 | .283 |
| Physical aggression age 5 | Mean (SD) | 5.7 (4.9) | 6.0 (4.9) | .679 | .864 |
| Community violence age 3 | Mean (SD) | 1.2 (2.3) | 1.5 (2.3) | .415 | .864 |
| Community violence age 5 | Mean (SD) | 1.1 (2.4) | 1.3 (2.4) | .624 | .864 |
| Arrangements age 3 | Mean (SD) | 0.7 (0.7) | 0.6 (0.7) | .452 | .864 |
| Arrangements age 5 | Mean (SD) | 0.1 (0.4) | 0.2 (0.6) | .339 | .819 |
| Regular bedtime age 3 | Mean (SD) | 2.4 (1.9) | 3.0 (2.2) | .037 | .283 |
| Regular bedtime age 5 | Mean (SD) | 1.9 (1.5) | 2.4 (1.8) | .070 | .315 |
| Bedtime routine age 3 | Mean (SD) | 2.2 (1.9) | 2.1 (1.9) | .805 | .864 |
| Bedtime routine age 5 | Mean (SD) | 2.4 (2.0) | 2.4 (1.9) | .777 | .864 |
| Maternal depression change age 3 | No | 2162 (98.1) | 41 (1.9) | .665 | .864 |
|  | Yes | 565 (97.8) | 13 (2.2) |  |  |
| Maternal depression change age 5 | No | 2169 (98.1) | 41 (1.9) | .815 | .864 |
|  | Yes | 555 (97.9) | 12 (2.1) |  |  |
| Separations age 3 | Mean (SD) | 0.2 (0.6) | 0.2 (0.6) | .866 | .866 |
| Separations age 5 | Mean (SD) | 0.3 (0.7) | 0.3 (0.7) | .567 | .864 |
| Moves age 3 | Mean (SD) | 0.6 (0.8) | 0.7 (1.0) | .776 | .864 |
| Moves age 5 | Mean (SD) | 0.7 (0.8) | 0.6 (0.9) | .801 | .864 |
| Jobs age 3 | Mean (SD) | 1.5 (1.0) | 1.3 (1.0) | .265 | .746 |
| Jobs age 5 | Mean (SD) | 1.3 (0.9) | 1.0 (0.8) | .076 | .315 |

**Supplementary Table S9.** **Missing data analysis for the Externalising behaviours age 15 outcome variable.** Comparisons for continuous data use a Kruskal Wallis and for discrete data a chi-squared test. No difference survives correction for multiple comparisons

|  | 1. | 2. | 3. | 4. | 5. | 6. | 7. | 8. | 9. | 10. | 11. | 12. | 13. | 14. | 15. | 16. | 17. |
| --- | --- | --- | --- | --- | --- | --- | --- | --- | --- | --- | --- | --- | --- | --- | --- | --- | --- |
| 1. Deprivation | - | .32^***^ | .21^***^ | .17^***^ | .25^***^ | .22^***^ | .09^*^ | .01 | .22^***^ | .10^***^ | .22^***^ | -.26^***^ | -.27^***^ | .05 | .14^***^ | -.11^**^ | -.09^*^ |
| 2. Threat |  | - | .14^***^ | .25^***^ | .27^***^ | .21^***^ | .13^***^ | .06^*^ | .41^***^ | .26^***^ | .25^***^ | -.19^***^ | -.20^***^ | .07^*^ | .14^***^ | -.08^**^ | -.08^*^ |
| 3. Stochasticity |  |  | - | .04 | .68^***^ | .08^*^ | .08^*^ | .01 | .10^**^ | .04 | .05 | -.06^*^ | -.05 | .00 | .00 | -.03 | -.02 |
| 4. Volatility |  |  |  | - | .76^***^ | .09^*^ | .06 | .05 | .15^***^ | .11^**^ | .11^**^ | -.19^***^ | -.14^***^ | .01 | .10^*^ | -.01 | -.19^***^ |
| 5. Overall unpredictability |  |  |  |  | - | .08^*^ | .08^*^ | .00 | .17^***^ | .13^**^ | .12^**^ | -.17^***^ | -.13^**^ | .01 | .08 | -.04 | -.16^***^ |
| 6. Internalising  age 5 |  |  |  |  |  | - | .42^***^ | .23^***^ | .48^***^ | .30^***^ | .18^***^ | -.15^***^ | -.14^***^ | .03 | .01 | -.01 | .00 |
| 7. Internalising  age 9 |  |  |  |  |  |  | - | .35^***^ | .27^***^ | .66^***^ | .23^***^ | -.07^**^ | -.07^*^ | -.04 | -.01 | .05 | .04 |
| 8. Internalising  age 15 |  |  |  |  |  |  |  | - | .23^***^ | .31^***^ | .54^***^ | -.03 | -.02 | -.02 | -.02 | .00 | .01 |
| 9. Externalising  age 5 |  |  |  |  |  |  |  |  | - | .49^***^ | .37^***^ | -.17^***^ | -.16^***^ | .05 | .08^**^ | -.05 | -.06^*^ |
| 10. Externalising  age 9 |  |  |  |  |  |  |  |  |  | - | .47^***^ | -.14^***^ | -.13^***^ | .03 | .12^***^ | -.04 | -.07^*^ |
| 11. Externalising  age 15 |  |  |  |  |  |  |  |  |  |  | - | -.14^***^ | -.14^***^ | .10^***^ | .15^***^ | -.13^***^ | -.11^***^ |
| 12. SES age 9 |  |  |  |  |  |  |  |  |  |  |  | - | .74^***^ | -.06^*^ | -.11^***^ | .07^**^ | .09^***^ |
| 13. SES age 15 |  |  |  |  |  |  |  |  |  |  |  |  | - | -.06^*^ | -.12^***^ | .08^**^ | .09^***^ |
| 14. Nr of sexual partners |  |  |  |  |  |  |  |  |  |  |  |  |  | - | .26^***^ | -.74^***^ | -.13^***^ |
| 15. Nr of date partners |  |  |  |  |  |  |  |  |  |  |  |  |  |  | - | -.23^***^ | -.56^***^ |
| 16. Age at 1^st^ sex |  |  |  |  |  |  |  |  |  |  |  |  |  |  |  | - | .16^***^ |
| 17. Age at 1^st^ date |  |  |  |  |  |  |  |  |  |  |  |  |  |  |  |  | - |

**Supplementary Table S10.** **Bivariate Pearson’s correlations between primary variables in the unimputed dataset.**

* p < .05 ** p < .01 *** p < .001

|  | **Latent variables / Outcomes** |  | **Indicators / Predictors** | **B** | **95% CI** | **se** | **z** | **p** | **β** |
| --- | --- | --- | --- | --- | --- | --- | --- | --- | --- |
| Measurement model | **Reproductive effort** | **=~** | **Age at 1st sexual intercourse** | **-0.215** | **[-0.291, -0.139]** | **0.039** | **-5.52** | **< .001** | **-.219** |
|  | **Reproductive effort** | **=~** | **Age at 1st date** | **-0.698** | **[-0.804, -0.591]** | **0.054** | **-12.84** | **< .001** | **-.710** |
|  | **Reproductive effort** | **=~** | **Number of sexual partners** | **0.159** | **[0.090, 0.229]** | **0.035** | **4.49** | **< .001** | **.162** |
|  | **Reproductive effort** | **=~** | **Number of date partners** | **0.687** | **[0.569, 0.805]** | **0.060** | **11.42** | **< .001** | **.699** |
| Structural model | Reproductive effort | ~ | Stochasticity | 0.001 | [-0.060, 0.062] | 0.031 | 0.04 | .972 | .001 |
|  | **Reproductive effort** | **~** | **Volatility** | **0.186** | **[0.118, 0.253]** | **0.035** | **5.38** | **< .001** | **.183** |
|  | Internalising behaviours | ~ | Reproductive effort | -0.022 | [-0.074, 0.031] | 0.027 | -0.80 | .424 | -.022 |
|  | Internalising behaviours | ~ | Stochasticity | -0.029 | [-0.080, 0.021] | 0.026 | -1.13 | .259 | -.029 |
|  | **Internalising behaviours** | **~** | **Volatility** | **0.086** | **[0.037, 0.136]** | **0.025** | **3.41** | **.001** | **.086** |
|  | Indirect effect of stochasticity on internalising |  |  | 0.000 | [-0.002, 0.002] | 0.001 | -0.03 | .974 | .000 |
|  | Total effect of stochasticity on internalising |  |  | -0.029 | [-0.080, 0.021] | 0.026 | -1.13 | .257 | -.029 |
|  | Indirect effect of volatility on internalising |  |  | -0.004 | [-0.015, 0.007] | 0.005 | -0.74 | .459 | -.004 |
|  | **Total effect of volatility on internalising** |  |  | **0.082** | **[0.034, 0.130]** | **0.025** | **3.34** | **.001** | **.082** |
|  | **Externalising behaviours** | **~** | **Reproductive effort** | **0.129** | **[0.074, 0.184]** | **0.028** | **4.59** | **< .001** | **.131** |
|  | Externalising behaviours | ~ | Stochasticity | -0.013 | [-0.059, 0.033] | 0.023 | -0.56 | .573 | -.013 |
|  | Externalising behaviours | ~ | Volatility | 0.039 | [-0.012, 0.090] | 0.026 | 1.50 | .133 | .039 |
|  | Indirect effect of stochasticity on externalising |  |  | 0.000 | [-0.008, 0.008] | 0.004 | 0.04 | .971 | .000 |
|  | Total effect of stochasticity on externalising |  |  | -0.013 | [-0.059, 0.033] | 0.024 | -0.55 | .580 | -.013 |
|  | **Indirect effect of volatility on externalising** |  |  | **0.024** | **[0.009, 0.039]** | **0.008** | **3.18** | **.009** | **.024** |
|  | **Total effect of volatility on externalising** |  |  | **0.063** | **[0.013, 0.113]** | **0.025** | **2.49** | **.013** | **.063** |

**Supplementary Table S11.** **Parameter estimates of the latent mediator model.** We also report under the z label values of Wald tests (and their p values). Parameters statistically significantly different from 0 are highlighted in bold.

**
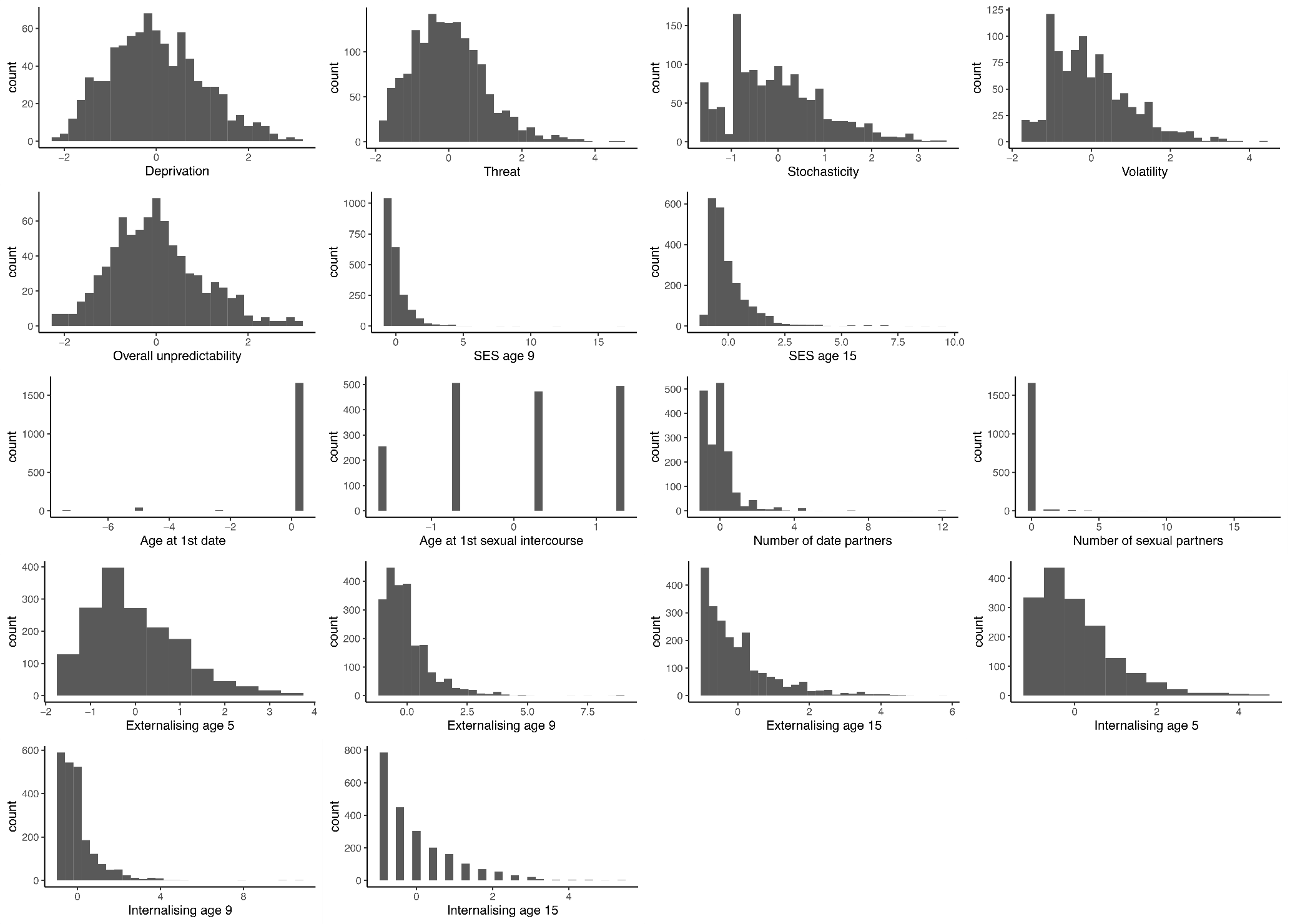
Supplementary Figure S1.** **Histograms showing the distributions of primary variables.** Note that these plots show the distribution of the standardized variables used for the main linear models, which is why the problem behaviour variables do not start at 0.

**
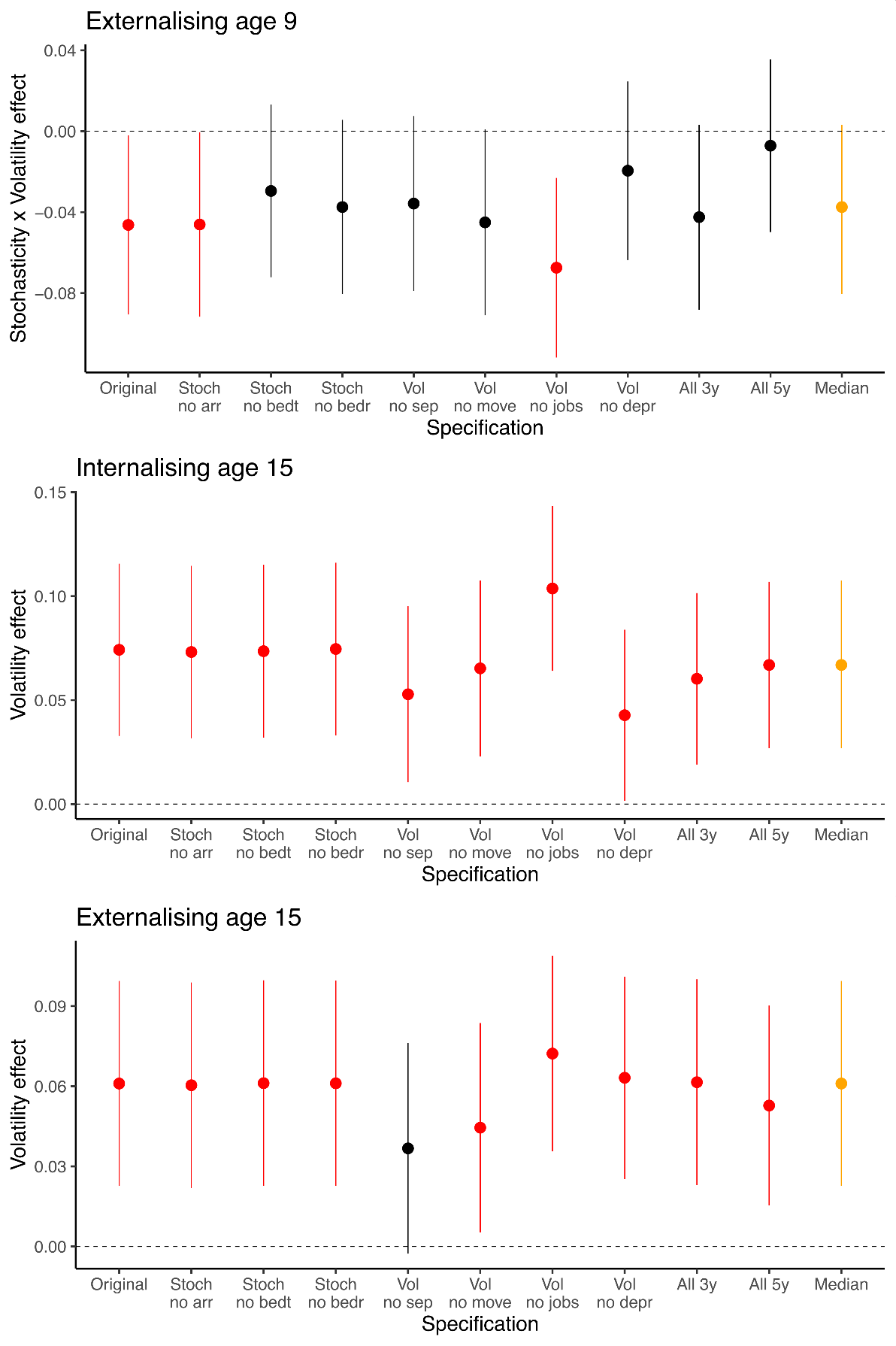
Supplementary Figure S2.** **Sensitivity analyses for alternative model specifications.** First column is the original model, reported in the main text, following columns indicate the same effect in the same model, with different ways of creating the stochasticity and volatility composites. Either one indicator was dropped, or only indicators for one age were used. Dots indicate the effect size, error bars indicate the 95% CI. Last column shows the median effect and 95% CI across specifications.


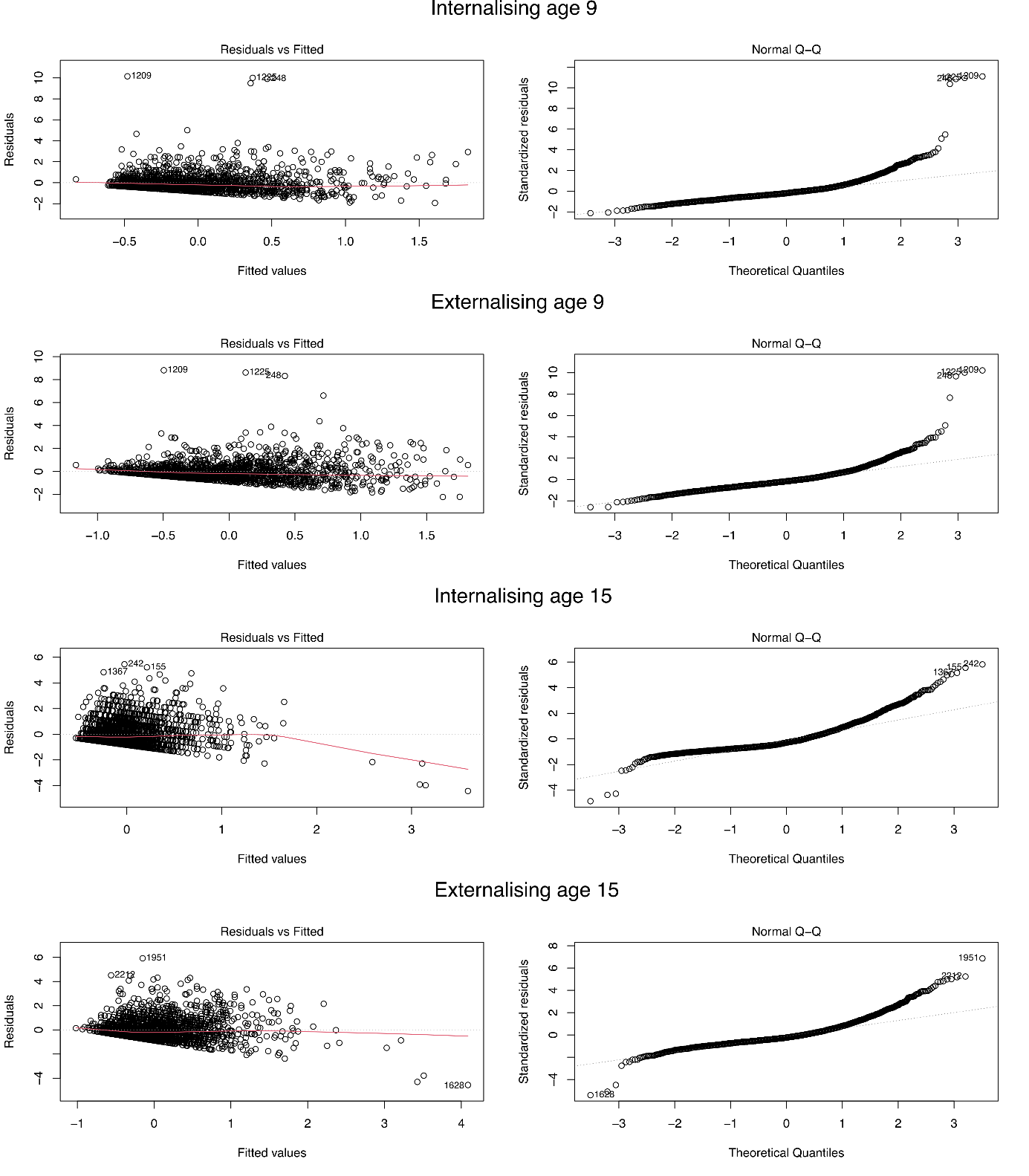
**Supplementary Figure S3.** **Plots of model residuals against fitted values, and Q-Q plots of standardized residuals for each of the 4 linear models reported in the main text.** Slight homoscedasticity and deviations from normality are detectable at the extremely high end of the outcome variables. Note however, that in large sample size settings, such as ours, the normality assumption can be largely ignored, and corrections for non-normality can even bias estimates (Schmidt & Finan, 2018).

Schmidt, A. F., & Finan, C. (2018). Linear regression and the normality assumption. *Journal of Clinical Epidemiology*, *98*, 146–151. <https://doi.org/10.1016/j.jclinepi.2017.12.006>


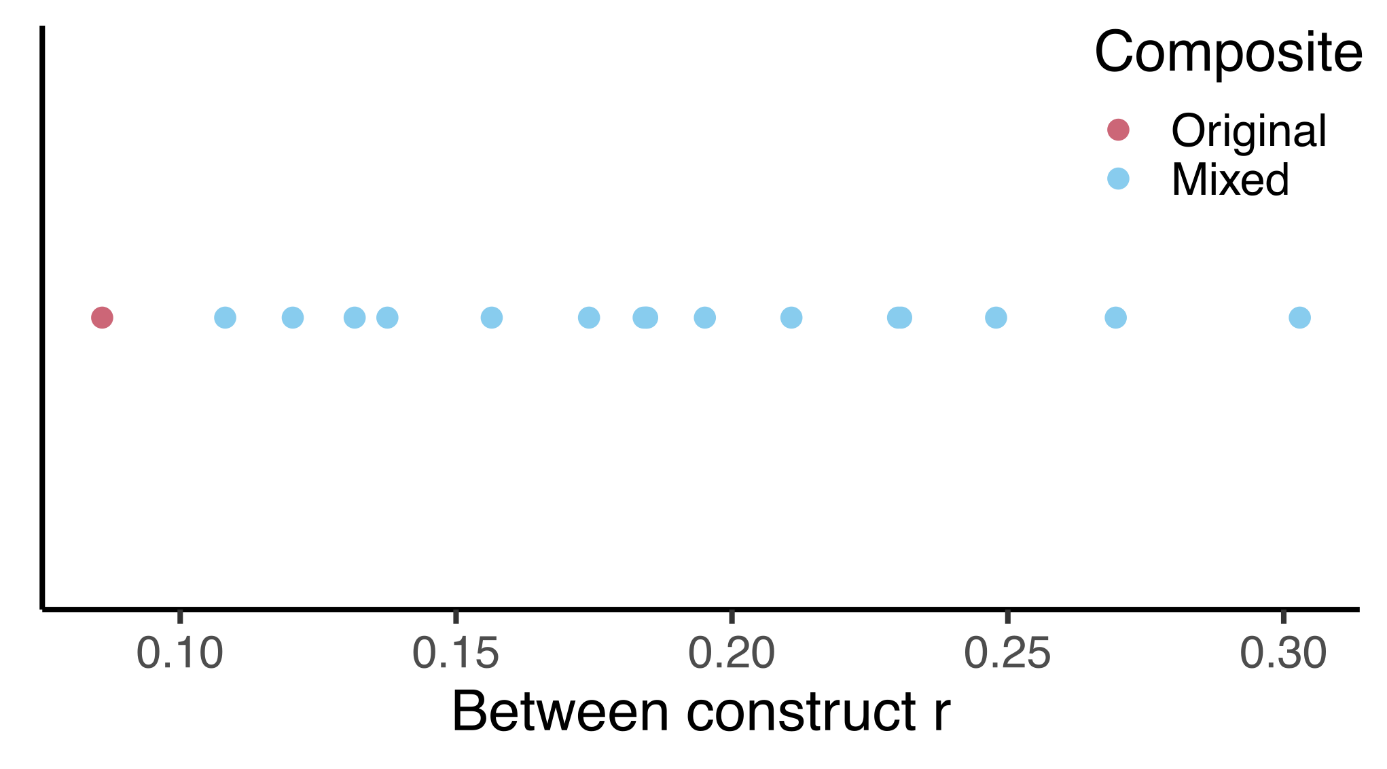
**Supplementary Figure S4.** **Original and mixed unpredictability composite correlations.** Between construct correlation of the original, theory-driven unpredictability composites, reflecting stochasticity and volatility (in red), and composites reflecting other possible combinations of our 7 indicators, mixing stochastic and volatile unpredictability (in blue).


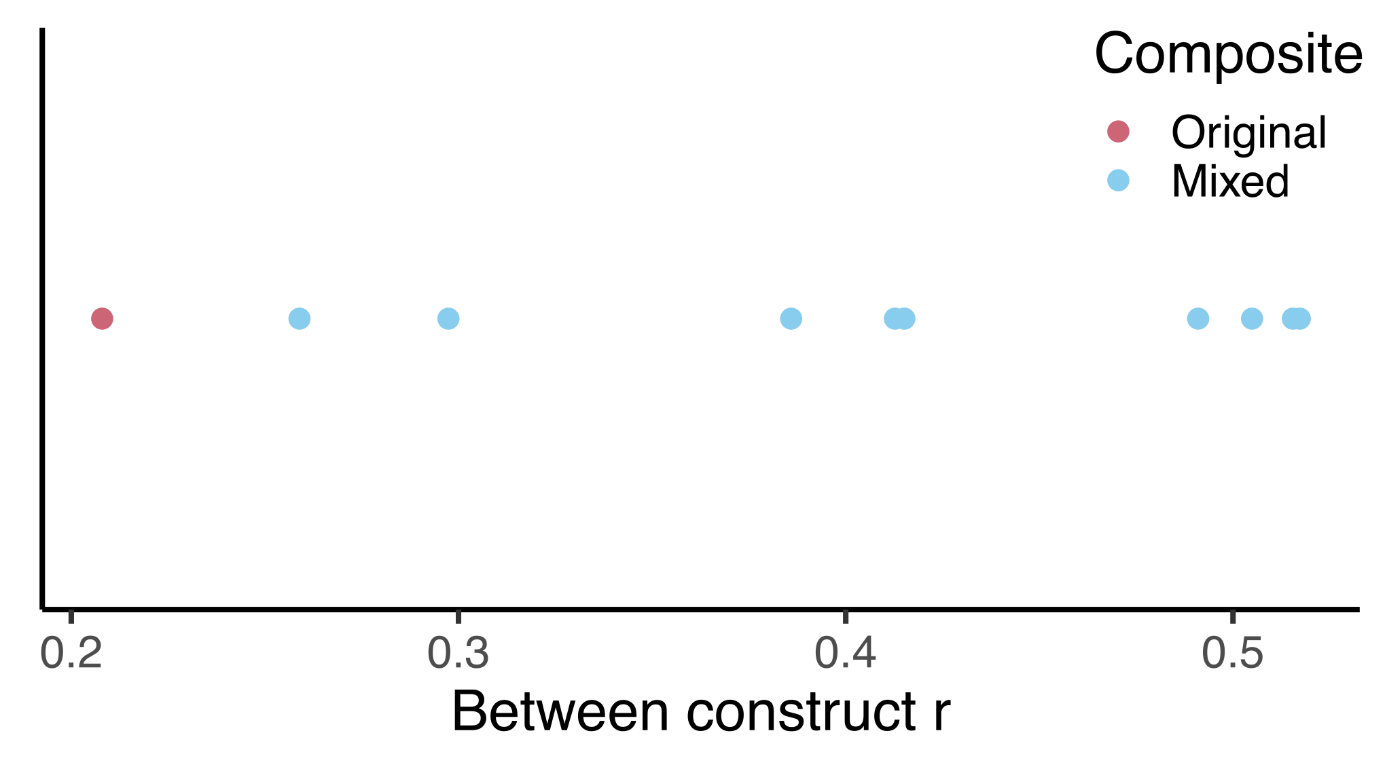
**Supplementary Figure S5.** **Original and mixed threat and deprivation composite correlations.** Between construct correlation of the original, theory-driven composites, reflecting deprivation and threat (in red), and composites reflecting other possible combinations of the 6 indicators, mixing deprivation and threat (in blue).
